# The cryo-EM structure of the human ERAD retrotranslocation complex

**DOI:** 10.1126/sciadv.adi5656

**Published:** 2023-10-13

**Authors:** Bing Rao, Qian Wang, Deqiang Yao, Ying Xia, Wenguo Li, Yuming Xie, Shaobai Li, Mi Cao, Yafeng Shen, An Qin, Jie Zhao, Yu Cao

**Affiliations:** ^1^ Department of Orthopaedics, Shanghai Key Laboratory of Orthopaedic Implant, Shanghai Ninth People's Hospital, Shanghai Jiao Tong University School of Medicine, Shanghai 200011, China.; ^2^ Shanghai Institute of Precision Medicine, Shanghai Ninth People’s Hospital, Shanghai Jiao Tong University School of Medicine, 115 Jinzun Road, Shanghai 200125, China.; ^3^ State Key Laboratory of Oncogenes and Related Genes, Ren Ji Hospital, Shanghai Jiao Tong University School of Medicine, Shanghai 200127, China.; ^4^ College of Basic Medical Sciences, Shanghai Jiao Tong University School of Medicine, Shanghai 200025, China.; ^5^ Department of Orthopaedics, Shanghai Frontiers Science Center of Degeneration and Regeneration in Skeletal System, Shanghai Ninth People's Hospital, Shanghai Jiao Tong University School of Medicine, Shanghai 200011, China.

## Abstract

Endoplasmic reticulum–associated degradation (ERAD) maintains protein homeostasis by retrieving misfolded proteins from the endoplasmic reticulum (ER) lumen into the cytosol for degradation. The retrotranslocation of misfolded proteins across the ER membrane is an energy-consuming process, with the detailed transportation mechanism still needing clarification. We determined the cryo-EM structures of the hetero-decameric complex formed by the Derlin-1 tetramer and the p97 hexamer. It showed an intriguing asymmetric complex and a putative coordinated squeezing movement in Derlin-1 and p97 parts. With the conformational changes of p97 induced by its ATP hydrolysis activities, the Derlin-1 channel could be torn into a “U” shape with a large opening to the lipidic environment, thereby forming an entry for the substrates in the ER membrane. The EM analysis showed that p97 formed a functional protein complex with Derlin-1, revealing the coupling mechanism between the ERAD retrotranslocation and the ATP hydrolysis activities.

## Teaser

Derlin-1 can associate with p97 and use its ATPase activities to change the channel conformation for ERAD retrotranslocation.

## INTRODUCTION

The endoplasmic reticulum (ER) is a pivotal organelle in protein homeostasis, which mediates the production and quality control of newly produced proteins. In defective proteins, such as those with destructive mutations and folding issues, ER-associated degradation (ERAD) is initiated, whereby the proteins are directed to the cytosolic proteasome for degradation (*1*–*3*). This process of protein transportation across the ER membrane, termed retrotranslocation, is the rate-limiting step due to the energy required to breach the lipid bilayer. Multiple molecular systems were identified to facilitate retrotranslocation (*4*, *5*). Structural studies of the yeast ERAD complex, formed by Der1, Hrd1, Hrd3, and Yos9, have suggested a “membrane-thinning” mechanism where the ER membrane is distorted to allow the protein substrate to penetrate through (*6*, *7*). In humans, Derlin-1, a homolog of yeast Der1, was found to form a tetrameric channel with a central tunnel ~10 Å wide, indicating a protein channel mechanism for retrotranslocation (*8*). Those precedent findings provide insights into the transmembrane process of retrotranslocation. However, the energy-coupling mechanism of retrotranslocation and the regulation of the retrotranslocation machinery’s open/close state transition remain to be elucidated.

Transitional ER adenosine triphosphatase (ATPase) (VCP/p97 in vertebrates and Cdc48 in yeast) was identified as the primary energy supplier for ERAD (*9*, *10*) and has been shown to power the defolding of ubiquitinated proteins via its interactions with the heterodimer Ufd1-Npl4 (*11*–*14*) and the deglycosylation through its interaction with N-glycanase 1 (*15*, *16*). Cdc48/p97 forms a stable homohexameric ring, and each subunit has two adenosine triphosphate (ATP) hydrolysis centers (*17*–*20*). As a multifunctional platform, Cdc48/p97 was found to bind with various proteins involved in ER functions (*21*), Golgi homeostasis (*22*), endosomal trafficking (*23*), etc. Human Derlin-1 was reported to bind to the N-terminal domain (NTD) of p97 via its SHP (suppressor of high-copy PP1 protein) box, which is essential for the extraction of a misfolded truncated fragment of α-1 antitrypsin (NHK) (*24*, *25*). In yeast, the well-studied Derlin-1 homolog, Der1, might not interact with Cdc48/p97 due to lacking a C-terminal SHP box (*26*, *27*). However, Dfm1, another yeast homolog for human Derlin-1 with two SHP boxes at the C terminus, could bind with Cdc48 to mediate the extraction of ERAD integral membrane protein substrates (*28*–*30*). Nevertheless, the mechanism by which p97 drives the ERAD retrotranslocation process is yet to be well defined, and the components and architecture of the retrotranslocation machinery are poorly understood.

To better comprehend the coupling mechanism of p97 ATPase and protein transportation across the ER membrane, we purified human Derlin-1 in complex with p97 at different ATP hydrolysis states and determined their cryo–electron microscopy (EM) structures. The profound conformational changes of the ERAD retrotranslocation channel in response to different catalytic states of p97 provide mechanistic implications for the retrotranslocation energized by ATPase activities.

## RESULTS

### Cryo-EM analysis on Derlin-1 and VCP/p97 complex

**Fig. 1.**
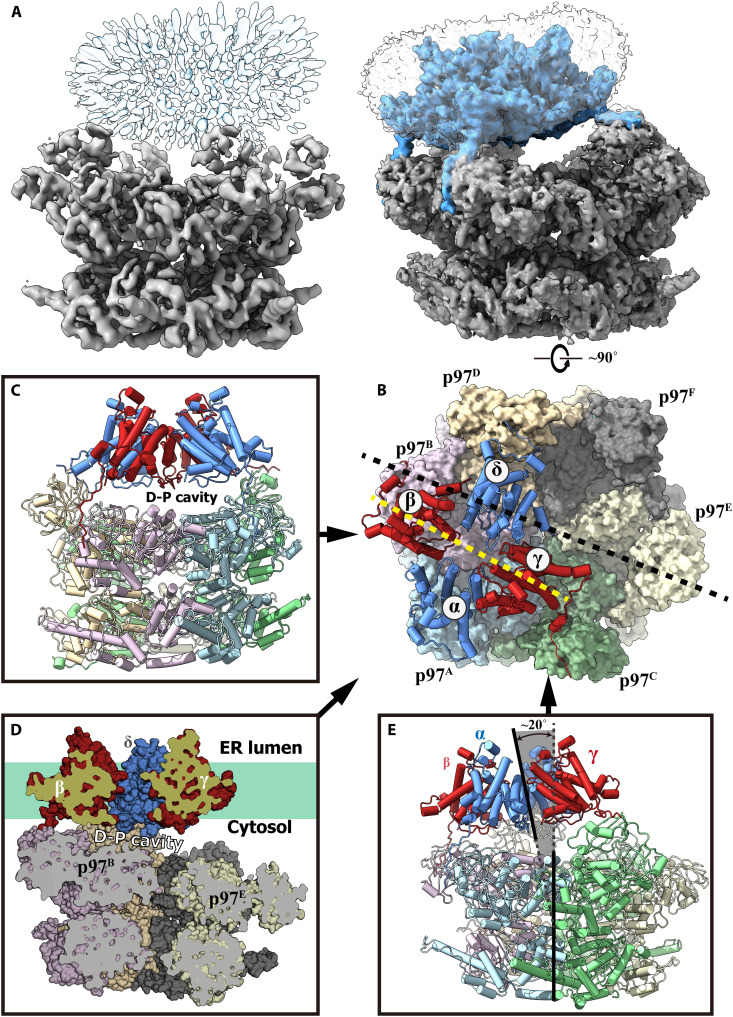
The overall architecture of the decameric complex of human Derlin-1 and p97. (**A**) Cryo-EM structures of the complexes of the full-length Derlin-1 with p97 (left) and Derlin-1 (Δ215–239) with p97 (Δ1–20) (right). For both complexes, the electron density map corresponding to p97 was shown in gray, and the unsolved electron density for detergent was shown as a transparent map. For the Der^Δ^-p97^Δ^ complex, the electron density map corresponding to Derlin-1 (Δ215–239) was shown in blue. (**B**) Structural model of the Der^Δ^-p97^Δ^ complex. The structure was shown as a surface model (for the p97 hexamer) combined with a cartoon model (for the Derlin-1 tetramer) and viewed from the lumen side of the ER membrane. The protomers in tetrameric Derlin-1 were designated as α, β, γ, and δ and colored blue (α and δ) and red (β and γ). The six p97 protomers were designated as p97^A-F^ and colored light blue (p97^A^), light pink (p97^B^), green (p97^C^), golden (p97^D^), light yellow (p97^E^), and gray (p97^F^). (**C** to **E**) Structural model of the Der^Δ^-p97^Δ^ complex viewed parallel to the ER membrane at the angles indicated by the arrows. (C) and (E) show the decamer as cartoon models, with the D-P cavity shown in (C) and the tilting angles between the central axis of the Derlin-1 tetramer and the p97 hexamer indicated in (E). (D) shows the central tunnel within the Der^Δ^-p97^Δ^ complex. The Der^Δ^-p97^Δ^ complex was shown as a surface model, with the two parts, Derlin-1, and p97, intersected independently with the planes indicated in (B).

The transient cotransfection of Expi293F cells with expression vectors for human Derlin-1 and VCP/p97 resulted in a stable complex, herein referred to as Der-p97, which was purified by twin-strep tag on Derlin-1 (fig. S1). Detergent screening and gel filtration separation indicated that Der-p97 existed as monodispersed single particles in glyco-diosgenin (GDN). Electron microscope imaging of Der-p97, collected at 300 kV using a Titan Krios transmission electron microscope (FEI) and processed using RELION3 and cryoSPARC (*31*, *32*), identified a hexameric p97 bound with a large electron density at the surface, composed of six NTDs of p97 (fig. S2). However, the 3D classification and refinement failed to resolve the electron density map for the transmembrane helices of Derlin-1, although the overall shape and size of the unsolved electron density matched the Derlin-1 tetramer with a detergent shell (Fig. 1A and fig. S2). The image analysis of the electron density for Der-p97 showed that various conformations might exist for the architecture of Der-p97 (fig. S2), which could result from the high flexibility of the interaction domain of the Der-p97 complex. It has been reported that the truncation of the N-terminal nonstructural 20–amino acid residues of p97 NTD increases the affinity of p97 with the C-terminal SHP box of Derlin-1 (amino acids 241 to 248) (*25*), which was missing in the cryo-EM structure of Derlin-1 due to its flexibility (*8*). To improve the signal-to-noise ratio of EM data by stabilizing the conformation of the Der-p97 complex, we implemented a series of Derlin-1 loop deletion constructs with varying deletion lengths and regions between the TM6 helix and the SHP box. Most of the constructs resulted in lower yields or produced EM images with unresolved Derlin-1. However, a few constructs generated yields comparable to the wild-type (WT) Derlin-1/p97 complex. Among these, the Derlin-1^Δ215–239^ construct exhibited the highest yield and provided the best resolution at the electron density corresponding to Derlin-1 in the EM map. Hence, for the subsequent EM analysis in this study, we used the constructs of Derlin-1^Δ215–239^ and p97^Δ1–20^, herein referred to as Der^Δ^-p97^Δ^. The cell-based ERAD assay showed that coexpression of Derlin-1^Δ215–239^ and p97^Δ1–20^ largely retained the ability of aberrant protein cleanup (fig. S3E). Subsequent cryo-EM analysis of Der^Δ^-p97^Δ^ showed improved homogeneity in the 2D classification (fig. S3). The three-dimensional (3D) classification and refinement generated an EM density map with an overall resolution of 3.7 Å and a local resolution ranging from 2.5 Å to 4.5 Å at the core region of the complex (fig. S4). To further investigate the conformational change upon the ATP hydrolysis of p97, the Der^Δ^-p97^Δ^ complex was supplemented with the nonhydrolysis analogs ADP·BeF_x_ and ATP·BeF_x_ before cryo-EM analysis. Following similar data collection and processing procedures, the corresponding cryo-EM maps were generated at resolutions of 3.61 to 4.51 Å (figs. S6 and S8 and Table 1).

**Table 1.** Cryo-EM data collection, processing, model refinement, and validation statistics.

	Der^Δ^-p97^Δ^ apo (PDB: 7Y4W EMDB-33608)	Der^Δ^-p97^Δ^/ADP·BeF_x_ (PDB: 7Y53 EMDB-33611)	openDer^Δ^-p97^Δ^/ATP·BeF_x_ (PDB: 7Y59 EMDB-33613)
**Data collection and processing**			
Microscope	Titan Krios G3i	Titan Krios G3i	Titan Krios G3i
Detector	Gatan K3 camera	Gatan K3 camera	Gatan K3 camera
Nominal magnification	81,000×	81,000×	81,000×
Voltage (kV)	300	300	300
Energy filter slit width	15 eV	15 eV	15 eV
Pixel size (Å/pixel)	1.1	1.1	1.1
Electron exposure (e^−^/Å^2^)	50	50	50
Defocus range (μm)	−1.0 to −2.5	−1.0 to −2.5	−1.0 to −2.5
Symmetry imposed	C1	C1	C1
Number of movies	22,808	7,052	10,883
Final particle numbers	183,316	248,684	62,905
Map resolution (Å)	3.67	3.61	4.51
Fourier shell correlation threshold	0.143	0.143	0.143
Map sharpening B factor (Å^2^)	−81.7	−81.4	−169.0
**Model building and refinement**			
Model composition			
Protein residues	5365	5362	5365
Ligands	4	12	12
RMSDs from ideal			
Bond lengths (Å)	0.002	0.003	0.003
Bond angles (°)	0.598	0.672	0.618
**Validation**			
All-atom clashscore	8.77	9.97	16.20
Rotamer outliers (%)	0.00	0.00	0.00
Ramachandran plot			
Favored (%)	91.44	90.95	91.50
Allowed (%)	8.11	8.63	8.09
Outlier (%)	0.45	0.41	0.41
			

### The assembly of the Der^Δ^-p97^Δ^ heterodecameric complex

The Der^Δ^-p97^Δ^ heterodecameric complex consists of six p97 molecules and four Derlin-1 molecules, with a molecular weight above 0.6 MDa (Fig. 1B). Both the Derlin-1 tetramer and the p97 hexamer within the complex largely retain their respective structures as determined individually (Fig. 1, B and C). However, the C6 symmetry of p97 and the C2 symmetry of Derlin-1 (*8*, *20*, *33*) is disrupted within the Der^Δ^-p97^Δ^ complex. The binding between Derlin-1 and p97 is mainly mediated by the interaction of the Derlin-1 SHP boxes and p97 NTDs, as per a previous study using crystallography (*25*). As the stoichiometry of Derlin-1 and p97 within the decamer is unequal (Derlin-1:p97 = 4:6), only four of the six p97 protomers (p97^A–D^) have their NTDs bound with Derlin-1, and the NTDs of the remaining two protomers (p97^E^ and p97^F^) are unoccupied. This misalignment between the Derlin-1 tetramer and the p97 hexamer results in an approximately 15 to 20 Å translation and an approximately 20° tilting between their central axes (Fig. 1E). As depicted in Fig. 1D, both the Derlin-1 tetramer and the p97 hexamer form a channel at their respective pseudosymmetry axes. Despite the atypical geometry of the Der^Δ^-p97^Δ^ complex, these two channels are connected to a large cavity between Derlin-1 and p97 (D-P cavity) to form a long, continuous ERAD substrate pathway extending from the ER lumen to the opening at the C-terminal surface of p97 (Fig. 1D).

### The different conformations of p97 NTDs in contact with Derlin-1

**Fig. 2.**
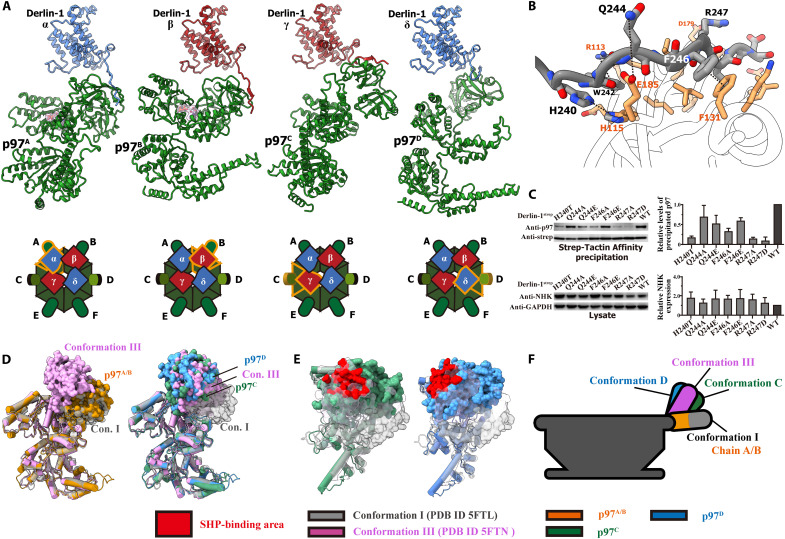
The interactions between the Derlin-1 channel and the p97 ring. (**A**) Top: Interacting pairs between Derlin-1 and p97. From left to right: Protomer pairs α-A, β-B, γ-C, and δ-D. The ADPs identified in p97^A/B^ were shown as the stick and surface models. Bottom: Cartoon representation of the organization of the complex, with the protomer pairs corresponding to the upper panel highlighted in orange. (**B**) Representative interactive network between the Derlin-1 SHP box and the p97 NTD. Both proteins were shown as stick models, with the Derlin-1 SHP box colored in gray-red-blue and the p97 NTD in orange-red-blue. (**C**) Interactions between the Derlin-1 channel and the p97 ring are critical for NHK cleanup. Top: Representative immunoblot results of the p97 pulled down by the strep-tagged Derlin-1 wild type and mutants (left) and the semiquantitative analysis independently repeated three times (right). Bottom: Representative immunoblot results of lysates of 293T cells coexpressing NHK, strep-tagged Derlin-1, and p97 (left) and the semiquantitative analysis independently repeated three times (right). The controls represented cotransfection of NHK with empty expression vectors. (**D**) Comparisons among the NTD conformations of p97^A/B/C/D^ with the Derlin-1–unbound p97 in its ADP- and ATP-binding states (conformations I and III, respectively). The p97 subunits in conformations I and III were superposed with p97^A/B^ (left) and p97^C/D^ (right), respectively. All the p97 protomers were shown as surface models for the NTD and cartoon models for the remaining part, with the color schemes as indicated below. (**E**) Differences of the p97^C/D^ NTD conformations and the Derlin-1 SHP-binding area on them. p97 in conformations I and III was superposed with p97^C^ (left) and p97^D^ (right), respectively. The SHP-binding areas on NTDs were highlighted in red. (**F**) Cartoon representations of the different p97 NTD conformations.

Previous structural studies on the conformational change of p97 showed that the NTDs of p97 remain “down” in the plane of the D1 hexamer (conformation I/II) when the D1 domains bind with adenosine diphosphate (ADP) and move to an “up” position (conformation III) upon ADP exchange with ATPγS (*20*). Analysis of the cryo-EM structures of Der^Δ^-p97^Δ^ revealed that p97 adapts to substantial conformational changes upon hydrolysis and cofactor exchange in its D1 and D2 domains. The tilting angle between the central axes of Derlin-1 and p97 causes two of the Derlin-1 protomers (Derlin-1^α/β^) to incline toward the p97 hexamer, thus lifting the other two Derlin-1 protomers (Derlin-1^γ/δ^) away from the p97 hexamer (Fig. 1E). In response, the four p97 NTDs bound with the Derlin-1 tetramer adopt different conformations to accommodate the tilted Derlin-1 tetramer (Fig. 2A), with the NTDs of p97^A/B^ in the down position as in conformation I and the NTDs of p97^C/D^ lifted to an up position to match the long distance between p97 and Derlin-1^γ/δ^, with the NTD of p97^C^ at a “higher” position and p97^D^ at a slightly “lower” position than the NTDs of p97 in conformation III (Fig. 2, D to F).

In the cryo-EM structure of Der^Δ^-p97^Δ^, the four Derlin-1 SHP box-p97 NTD interfaces, although in different overall conformations, exhibit the same local structures as previously reported (*25*). This association is maintained by multiple interactions, such as the electrostatic interaction between R247 of Derlin-1 and D179 of p97, the π-cation interaction between W242 of Derlin-1 and R113 of p97, and the π-π stacking between F246 of Derlin-1 and F131 of p97, as well as several hydrogen bonds such as the interaction between H240 of Derlin-1 and H115 of p97 (Fig. 2B). The binding between Derlin-1 SHP box and p97 NTD is essential for the formation and function of the complex in ERAD substrate cleanup. Mutations on H240, Q244, and F246 of Derlin-1 could disrupt their interactions with p97 and lead to higher accumulations of NHK in the cells (Fig. 2C), indicating the critical role of the Derlin-1/p97 complex in ERAD retrotranslocation. We performed a quantitative polymerase chain reaction (qPCR) analysis to rule out the possibility of NHK accumulations being influenced by non-ERAD factors, such as variations in the transfection or transcription efficacy of NHK. The qPCR results (fig. S9) demonstrated that the mRNA levels of NHKs cotransfected with p97 and Derlin-1 interface mutants were comparable or even lower when compared to those cotransfected with WT Derlin-1. This finding supports the functional relevance of Derlin-1/p97 interactions in ERAD retrotranslocation. Although mutations R247D and R247A can also disrupt the Derlin-1/p97 interaction, the corresponding NHK accumulations were comparable to those observed with WT Derlin-1. This observation can likely be attributed to their mRNA levels, notably lower than the NHK cotransfected with WT Derlin-1.

The structural analysis of the electron density map generated using the Der^Δ^-p97^Δ^ complex without ligand supplement (apo) revealed the presence of four sizeable nonproteinous electron density blobs at the catalytic center of the D1 domains from p97^A/B/E/F^ and ADP molecules fit nicely into them (Fig. 2A and fig. S9C). Additionally, the binding pockets of the remaining ATPase catalytic centers (all D2 domains and the D1 domains of p97^C/D^) were largely empty, with only some discontinuous residual densities. The two unoccupied NTDs from p97^E/F^ also demonstrated high flexibility within the complex. The major model determined from 3D classification showed the p97^E^ NTD in the down position and the p97^F^ NTD in a “partially up” position, yet not fully lifted into conformation III (fig. S5). Further 3D classification focusing on p97 revealed four subclasses, in which the p97^E/F^ NTDs were in different conformations, while the p97^A–D^ NTDs were essentially the same conformation as in the major class (figs. S4A and S5). The four subclasses were designated as “u-d,” “u-u,” “d-u,” and “d-d,” respectively, according to the up or down position of their NTD of p97^E/F^ (fig. S4A).

### The retrotranslocation pathway in the Der^Δ^-p97^Δ^ complex

**Fig. 3.**
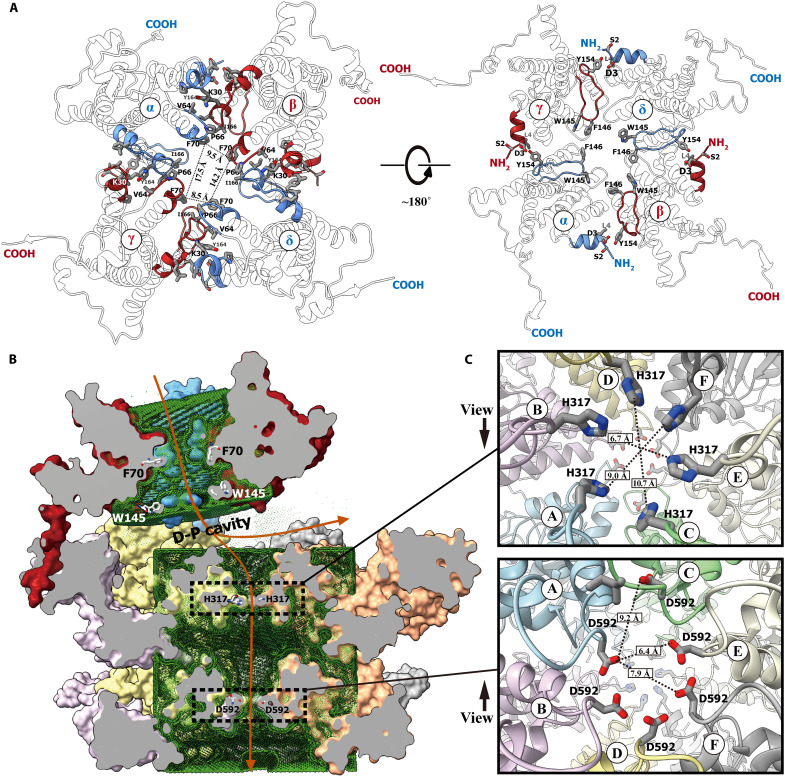
The substrate retrotranslocation tunnel in the Der^Δ^-p97^Δ^ complex. (**A**) Trans-ER membrane channel in the Der^Δ^-p97^Δ^ complex. The human Derlin-1 tetramer was shown as a cartoon model and viewed from the ER lumen (left) and cytoplasm (right). The major body of Derlin-1 was transparent with a black silhouette, and the central tunnel–surrounding part was colored blue (for protomers α/δ) and red (for protomers β/γ). The residues delineating the central tunnel were shown as stick models rendered by elements. (**B**) Whole central tunnel in the Der^Δ^-p97^Δ^ complex and the putative translocation pathway. The Derlin-1 channel and the p97 ring in the Der^Δ^-p97^Δ^ complex were shown as surface models and sliced along their pseudo-fourfold and pseudo-sixfold symmetry axis, respectively, to show the central tunnel. The central tunnels within the tetrameric Derlin-1 and hexameric p97, determined individually by HOLE, were shown as green dots. The residues gating the tunnel were shown as stick models, and the putative translocation pathway was indicated with the orange arrows. (**C**) Gating residues of the p97 central tunnel. The human p97 hexamer was shown as a cartoon model and viewed as indicated. The gating residues, H317 and D592, were shown as stick models rendered by elements. All the surface models in this figure were colored with the same scheme as in Fig. 1.

Previous studies using Hollow identified a sizeable central tunnel across the ER membrane in the human Derlin-1 channel (*8*), which connected to a large D-P cavity between the Derlin-1 tetramer and the p97 hexamer (Fig. 3B). The Derlin-1 tunnel was found to be quite large, with its C2 symmetry disrupted by the stretching of the p97 NTDs resulting in an uneven quadrilateral shape at its cross-section (Fig. 3, A and B), allowing ERAD substrates to pass through while maintaining their secondary structure. On the other end of the D-P cavity, the p97 central tunnel was observed to be relatively narrower than the Derlin-1 central tunnel (Fig. 3, B and C). As calculated by HOLE (*34*), the diameters at H317 and D592 of the p97 tunnel were about 4 Å and 4.6 Å, respectively. This near-symmetric arrangement of pore residues suggests a resting state of p97 without substrate binding since the structural studies on Cdc48 (the yeast homolog of p97) showed a dynamic staircase action upon the substrate binding in the central tunnel (*35*).

The tunnels within Derlin-1 and p97 were found to be misaligned, forming a broken line bend at the D-P cavity. Ubiquitination plays a crucial role in protein degradation, particularly in the ERAD process, where it occurs in conjunction with or immediately following retrotranslocation. Existing studies have demonstrated that the p97 hexamer and UFD1/NPL4 bind polyubiquitinated substrates and guide them into its central tunnel for subsequent delivery to the proteasome (*13*). This suggests the presence of a checkpoint between the retrotranslocation channel and the p97 ATPase. The D-P cavity, located between the exit of the Derlin-1 channel and the entrance of the p97 ring, emerges as a compelling candidate space for ERAD substrate ubiquitination.

### The coordinated actions of the Derlin-1 channel and p97 ATPase

**Fig. 4.**
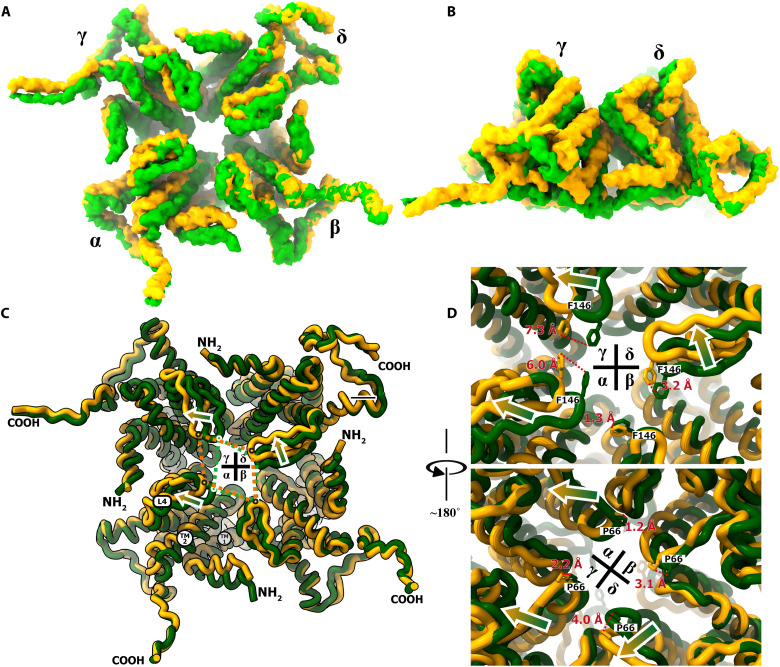
The coordinated operation of the Der^Δ^-p97^Δ^ complex revealed by 3D variability analysis. (**A** and **B**) Two major clusters with the substantial conformational difference viewed from the cytoplasm (A) and parallel to the ER membrane (B). The EM maps corresponding to the backbone of the Derlin-1 tetramer were shown as surface models in green (tightDer^Δ^-p97^Δ^ conformation) and yellow (Der^Δ^-p97^Δ^ apo conformation). (**C**) Structural superposition between the molecular models of the Derlin-1 tetramer from Der^Δ^-p97^Δ^ in loose apo conformation (yellow) and tightly closed conformation (green), with the protomer βs in two models aligned as the reference. The central dotted quadrilaterals showed the relative changes of the positions corresponding to the F70 residues from four Derlin-1 protomers. The secondary structures with the most notable displacements in the 3DVA (TM1, TM2, and loop L4) were indicated in protomer α. (**D**) Enlarged views on the central tunnel of the Derlin-1 tetramer at two opposite angles. The distances between the same residues in loose apo conformation (yellow) and tightly closed conformation (green) were shown as red lines.

The long central tunnel structure found in the Der^Δ^-p97^Δ^ complex suggests a function of a transporting-unfolding pipeline and the dynamic features of this molecular machinery. Our 3D classification of the EM particles revealed flexible structures of both Derlin-1 and p97, and 3D variability analysis (3DVA) was conducted to investigate the conformational changes in the Der^Δ^-p97^Δ^ complex (*36*). As shown in movie S1, the p97 hexamer twists as D1 and D2 oscillate, and with the same moving rhythm driven by the p97 NTD moving back and forth between the up and down conformations, the Derlin-1 channel changes its shape and size of its central tunnel and the distances from protomers γ to δ and α to β (Fig. 4C). Superposition of the structures using protomer β as a reference molecule revealed major conformational changes around the N terminus, the loop between transmembrane helices TM4 and TM5 (loop L4), as well as helices TM1 and TM2. These results showed that the Derlin-1 channel switches between a tightly closed state (tightDer^Δ^-p97^Δ^, state T) and a loose state (the major conformation shown in Figs. 1 to 3, state A). The root mean square deviation (RMSD) between states T and A was calculated as about 2.56 Å, suggesting a moderate conformation change. This dynamic change could result from a coupling between the p97 ATPase activities and the channel action during the substrate recognition and retrotranslocation or a passive motion in response to substrate passage.

### The open/close of Derlin-1 channels mediated by the ATPase activities of p97

To elucidate the dynamics of the Derlin-1 channel and the underlying driving mechanism, a range of ATP analogs were used to study the structures of the Der^Δ^-p97^Δ^ complex at different hydrolysis states. The analog ADP·BeF_x_ was first used to capture p97 at the initial state of ATP hydrolysis (*14*), and the cryo-EM analysis of the Der^Δ^-p97^Δ^/ADP·BeF_x_ complex yielded a structure that was similar to that of the apo Der^Δ^-p97^Δ^ complex, with an RMSD of approximately 0.5 Å (figs. S6 and S7). The nucleotide-binding sites were all occupied by an ADP molecule; however, the electron density of BeF_x_ was too weak for reliable assignment and modeling (fig. S11E). The NTDs of the p97^A/B/E/F^ protomers were in the down conformation, whereas the NTDs of the p97^C/D^ protomers were in an up conformation, even when bound with ADP, indicating the flexibility of p97^C/D^, which was able to adopt a “mixed” conformation combining a D1-D2 domain in conformation I and an NTD close to conformation III (Fig. 2F and fig. S7).

**Fig. 5.**
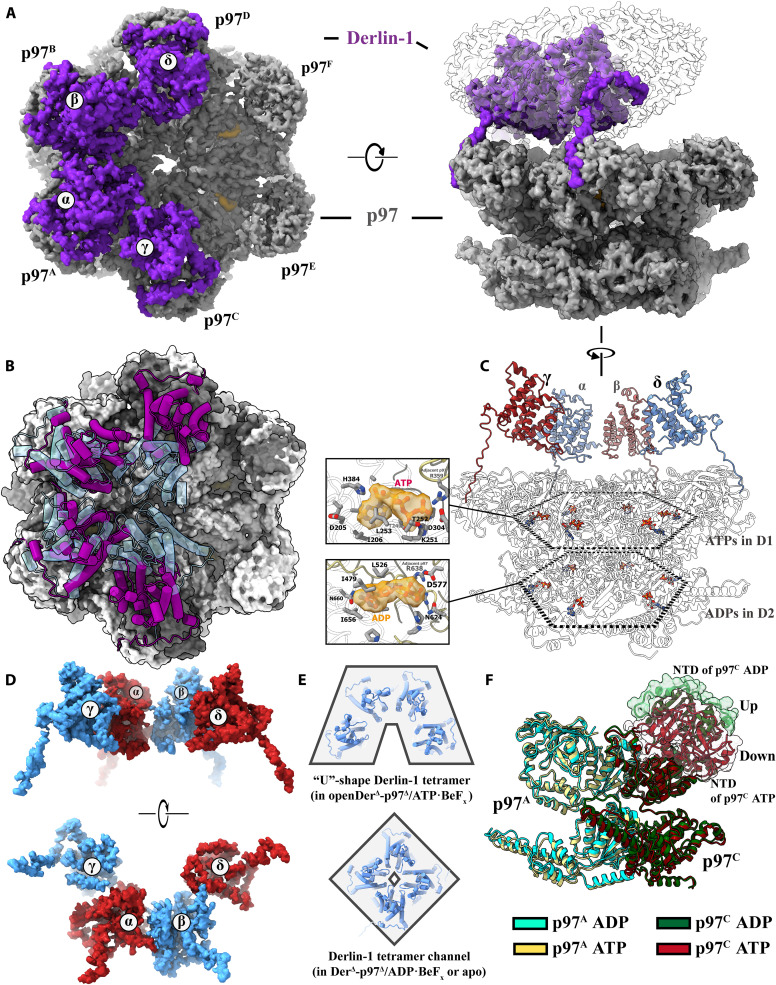
The openDer^Δ^-p97^Δ^/ATP·BeF_x_ complex and open conformation of Derlin-1. (**A**) EM map for Der^Δ^-p97^Δ^ bound with ATP·BeF_x_ in the open conformation viewed from the ER lumen (left) and parallel to the ER membrane (right). The electron density for Derlin-1 was shown in purple, p97 in gray, and ligands bound in orange. (**B**) Structural superposition between structures of the openDer^Δ^-p97^Δ^/ATP·BeF_x_ and Der^Δ^-p97^Δ^ apo complex. Derlin-1 was shown as cartoon model in purple for openDer^Δ^-p97^Δ^/ATP·BeF_x_ and in translucent blue for the Der^Δ^-p97^Δ^ apo. p97 was shown as a surface model colored in light gray for openDer^Δ^-p97^Δ^/ATP·BeF_x_ and dark gray for Der^Δ^-p97^Δ^ apo. (**C**) Cartoon representation of the openDer^Δ^-p97^Δ^/ATP·BeF_x_ complex. The Derlin-1 tetramer was colored blue (α and δ) and red (β and γ). p97 was colored white, with the ATP and ADP shown as stick models and colored by elements. The insets to the left of the model showed the binding pockets holding ATP and ADP in the D1 and D2 domain of p97^C^, respectively. (**D**) Cryo-EM map for the Derlin-1 tetramer in state U. The density maps corresponding to α and δ were colored in red, and the density maps corresponding to β and γ were colored in blue. (**E**) Comparison between the overall shape of the Derlin-1 tetramer in openDer^Δ^-p97^Δ^/ATP·BeF_x_ (upper, U shape) and the Der^Δ^-p97^Δ^ apo (lower, channel shape). Both models were shown as cartoon models. (**F**) Structural superposition among the p97^A/C^ protomers in the openDer^Δ^-p97^Δ^/ATP·BeF_x_ and the Der^Δ^-p97^Δ^ apo. The p97 protomers were shown as cartoon models and colored as the palette below the graph. The NTDs for p97^C^ were shown as translucent surface models and colored red for the openDer^Δ^-p97^Δ^/ATP·BeF_x_ conformation and green for the Der^Δ^-p97^Δ^ apo conformation.

By contrast, the Der^Δ^-p97^Δ^ complex assumes two distinct conformations upon addition of ATP·BeF_x_. Of the total selected particles, approximating 21.1% corresponded to a conformation analogous to that of the Der^Δ^-p97^Δ^ apo complex or Der^Δ^-p97^Δ^/ADP·BeF_x_ complex. In the other conformation represented by 20.8% of the particles, however, the Derlin-1^γ/δ^ interface is disrupted by displacement of the p97 NTDs, resulting in a “U”-shaped structure (state U) with a large opening toward the lipid environment of the ER membrane (Fig. 5, A and B). Notably, resolution of the Derlin-1 part within this open conformation is limited likely, due to increased local flexibility upon loss of the rigid channel structure (Fig. 5D). Nevertheless, the overall shape of the electron density and the C-terminal domain (CTD) associated with the p97 NTDs enabled reliable modeling. This opening, formed by the crevice between Derlin-1^γ/δ^, measures more than 30 Å in width (Fig. 5E). It appears to be caused by a conformational change of the NTDs of p97^C/D^ from the up to the down conformation, which leads to pulling Derlin-1^γ/δ^ away from each other and consequently breaking the γ-δ interface. In the p97 associated with the open Derlin-1 tetramer, all six protomers adopt a down conformation (Fig. 5F). Nonproteinous electron densities identified in the D1 domains fit ATP molecules well, while those in the D2 domain are smaller and thus accommodate ADP molecules (Fig. 5C and fig. S11E).

### The mechanistic implication of the ERAD retrotranslocation

**Fig. 6.**
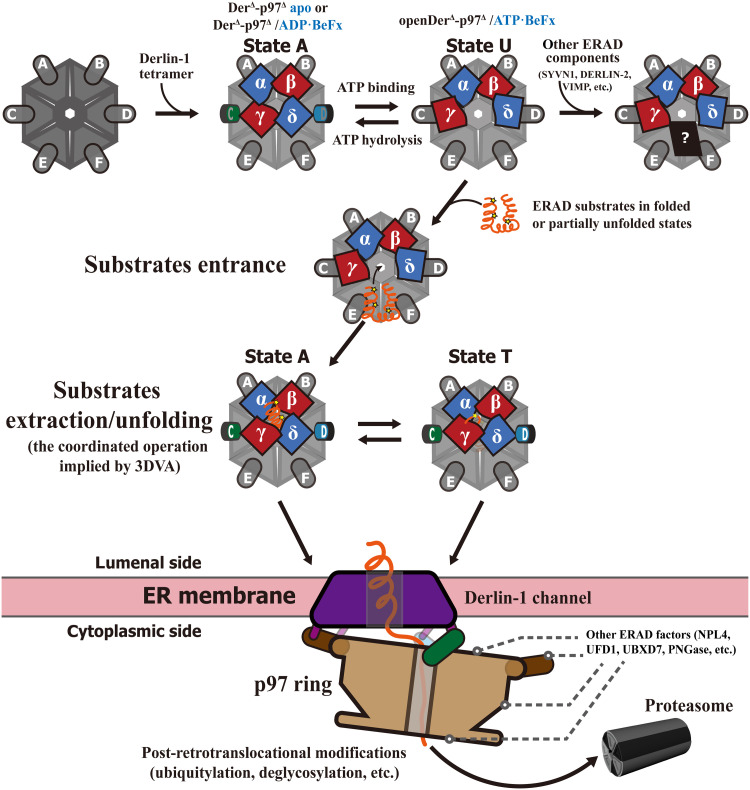
The working model for Derlin-1–mediated ERAD retrotranslocation. Upper cartoon representation with the top view of the Der^Δ^-p97^Δ^ complex: The formation of the Derlin-1/p97 complex and the channel operation by ATPase activities. The p97 hexamers were colored gray, with the NTD in gray for down conformation and green/sky blue for up conformation, respectively. The Derlin-1 tetramer was colored blue for protomers α/δ and red for β/γ. Lower cartoon representation with the side view of the Der^Δ^-p97^Δ^ complex in the ER membrane: The ERAD retrotranslocation machinery and its operational moment suggested by the cryo-EM analysis.

Our cryo-EM studies identified two major states for the human Derlin-1/p97 complex: state A, represented by the Der^Δ^-p97^Δ^ apo and Der^Δ^-p97^Δ^/ADP·BeF_x_ complex, and state U, represented by the openDer^Δ^-p97^Δ^/ATP·BeF_x_ complex. We additionally observed a tightly closed state T, shown as tightDer^Δ^-p97^Δ^ in 3DVA. These studies enabled us to propose an ERAD retrotranslocation model (Fig. 6). This model postulates that the process begins with the recruitment of p97 onto the Derlin-1 channel in the ER membrane, forming the Derlin-1/p97 complex in state A. In this complex, the p97^A/B^ NTDs remain mostly in conformation I (down), and their interactions with the SHP boxes of the protomers α and β of Derlin-1 play a role as “stator” to fix p97 on the Derlin-1 channel. At the same time, the NTDs of p97^C/D^ are relatively flexible upon ATP hydrolysis in their D1 domains and thus act as “rotors” in driving the conformational change in the Derlin-1 channel. This leads to a transformation between states A and T, whereby an intramolecular rearrangement of the transmembrane helices occurs, resulting in a twisting or squeezing movement of the central tunnel. The NTDs of p97^C/D^ could even cause a disruption of the Derlin-1 γ/δ interface, leading the complex to state U, with a side-open Derlin-1 channel, which enables the Derlin-1 channel to capture even larger substrates, such as proteins retaining their tertiary structures or partially unfolded. The relatively smaller actions in the state A to T transition may serve to extract the substrate from the ER membrane. Subsequently, the Derlin-1 channel presents the protein substrates to the central tunnel of p97. Additional ERAD factors may be recruited to p97 for further processing, such as unfolding, ubiquitylation, and deglycosylation.

To further explore the conformational changes of Derlin-1 caused by p97 binding, we conducted a superposition analysis of Derlin-1 structures from the channel-only state [Protein Data Bank (PDB) ID 7ZCB] (*8*), state T, and the Der^Δ^-p97^Δ^ apo complex (fig. S10). We did not include the state A and Der^Δ^-p97^Δ^ ADP complex in this comparison, as they do not exhibit substantial structural differences from the Der^Δ^-p97^Δ^ apo complex. The RMSDs of Derlin-1 between the Derlin-1 channel-only structure and the Derlin-1 tetramer in the Der^Δ^-p97^Δ^ apo and state T are 3.64 Å and 4.06 Å, respectively. As depicted in fig. S10, while the α and β subunits of Derlin-1 exhibited similar overall folding and arrangement of central tunnel residues, the δ and γ subunits in state T adopted a conformation closer to the center, resulting in a tighter overall structure. Conversely, the Derlin-1 channel in the channel-only state displayed a looser conformation compared to the Derlin-1 in the apo complex or state T. These observed conformational differences suggest that the Derlin-1 channel retains a C2-symmetric loose state when its CTD is free. However, upon capture by the p97 ring, the geometric conflict between the p97 NTD and Derlin-1 CTD causes the channel to adopt a slightly tighter state (Der^Δ^-p97^Δ^ ADP apo complex). Moreover, conformational changes in the p97 NTDs, driven by its ATPase activities, further tighten the Derlin-1 channel (state T) until extensive conformational changes in p97^C/D^ ultimately tear the channel into state U.

## DISCUSSION

The cryo-EM structures of the human ERAD retrotranslocation complex have been proposed in various molecular models, such as the membrane-thinning model and the full channel model. The membrane-thinning model, proposed based on the cryo-EM structures of the yeast Der1-Hrd1-Hrd3 complex (*6*), suggests that the membrane components Der1 and Hrd1 force the local ER membrane to be distorted and form a thinner region, which allows the ERAD substrate to pass through with minimal energy requirement. Moreover, the full channel model, proposed based on the EM structures of the human tetrameric Derlin-1 channel (*8*), suggests that the entire transmembrane channel formed by tetrameric Derlin-1 provides a pathway for large ERAD substrates. However, the energy consumption implied by this model was not studied mechanistically. In this context, the heterodecameric complex of human Derlin-1/p97 provides supportive information about the energy supply and alternative side-open conformation in the full channel model.

This study revealed the cryo-EM structure of the human ERAD retrotranslocation complex, comprising a Derlin-1 tetramer and a p97 hexamer. It showed an intriguing asymmetric complex between the two proteins and a coordinated squeezing movement in both Derlin-1 and p97 parts, implying the exit process of the substrate proteins. Furthermore, it revealed an unexpected conformational change in the Derlin-1 channel, where the channel was torn at the side wall to form an access between its central tunnel and the ER membrane.

Previous studies have linked p97 with various ERAD membrane components such as Hrd1/SYN1 (*37*), VIMP (*38*), Derlin-1 (*37*, *39*, *40*), and its closest homolog in yeast, Dfm1 (*28*, *41*). This study revealed an intriguing asymmetric complex between the Derlin-1 tetramer and the p97 hexamer, and the p97 NTD-Derlin-1 CTD interfaces act as gears in transmitting the “up-down” cycle of the p97 NTD induced by ATPase activity into the conformational changes of the Derlin-1 channel, as demonstrated by 3DVA. An unexpected discovery was the conformation of openDer^Δ^-p97^Δ^/ATP·BeF_x_ (state U). Here, the pulling action of p97 NTDs tore the Derlin-1 channel at the side wall to form an access between its central tunnel and the ER membrane, likely allowing the retrotranslocation complex to capture large protein substrates, such as partially unfolded proteins with some secondary structure retained. Additionally, this channel-breaking process may also be necessary for other ERAD components to join in, as the yeast Der-Hrd1 complex interface was embedded within the human Derlin-1 channel (*8*). The channel-breaking process presents a mechanistic implication of the ERAD complex assembly process where the Derlin-1 tetrameric ring is opened by p97, and the docking interface for Hrd1/SYVN1 or other proteins is thus exposed for their recruitment. This model agrees with previous findings that p97 could promote the binding of Hrd1/SYVN1 with Derlin-1 (*37*), and further efforts should be made to produce a larger ERAD complex with more components. Despite those supporting evidence for the conformational coupling between Derlin-1 and p97, care should be taken in reviewing the difference between the truncation Der^Δ^-p97^Δ^ structures and its natural states. Specifically, the longer CTD loop in the WT Derlin-1 could provide more flexibility in the natural state, which might limit the conformational change from state A to U to a moderate extent and result in a smaller open between Derlin-1 δ and γ promoters. Here, the flexibility leads to unsolved electron density map of the membrane-bound component, and thus, our observation on the full-length Derlin-1/p97 complex is limited to the variable geometry relationship between the Derlin-1 channel and p97 ring, as suggested by the 3D classification (fig. S2A). Therefore, additional studies, particularly cryo-EM structures of the WT Derlin-1/p97 complex, would be required to gain a more precise understanding of the interactions between p97 and Derlin-1.

While the precise structural details of the moment when substrate proteins pass through the Derlin-1/p97 complex remain elusive, our findings provide a mechanistic implication of the ERAD complex assembly process, and further structural studies focusing on the complex between the Derlin-1 channel and its substrates will be imperative to elucidate the entire retrotranslocation process. It is essential to acknowledge that the retrotranslocation process in ERAD is multifaceted and involves many factors beyond the Derlin-1/p97 complex. Other components, such as VIMP, UFD1/Npl4, PNGase, and HRD1/HRD3, play crucial roles in substrate recognition and processing throughout retrotranslocation. Thus, it is reasonable to speculate that the Derlin-1/p97 complex might be a core part within a larger assembly, encompassing these ERAD factors. As such, efforts aimed at elucidating the supracomplex structure of the retrotranslocation machinery hold great promise in advancing our understanding of ERAD.

## MATERIALS AND METHODS

### Protein expression and purification

The cDNA of human p97 [National Center for Biotechnology Information (NCBI) reference sequence: NM_007126.3] was cloned into the pcDNA3.4 vector with a C-terminal His tag or no tag, and the cDNA of human Derlin-1 (NCBI reference sequence: NM_024295.6) was cloned into a modified pcDNA3.4 vector with a PreScission Protease cleavage site and a C-terminal twin strep tag. Expi293F cells (Thermo Fisher Scientific, A14527) were cultured in chemically defined Union-293 medium (Union-Biotech, UP0050) at 37°C, supplied with 5% CO_2_. To coexpress the full-length Derlin-1–p97 complex, 1-liter Expi293F cells were transiently cotransfected with 0.5 mg of His-tagged pcDNA3.4-p97 and 0.5 mg of modified pcDNA3.4-Derlin-1 using PEI when the cell density reached 1.8 × 10^6^ cells per ml, and the transfected cells were cultured for 60 to 72 hours before harvest.

For the purification of the full-length Derlin-1–p97 complex, the transfected Expi293F cells were collected and resuspended in lysis buffer containing 20 mM Hepes (pH 7.5), 150 mM NaCl, 10% glycerol, 10 mM MgCl_2_, deoxyribonuclease (DNase) I (0.5 mg ml^−1^), and 1× protease inhibitor (MCE). The entire purification procedure was performed at 4°C. After sonication on ice, the membrane fraction was solubilized at 4°C for 2 hours with lysis buffer supplemented with 1% (w/v) lauryl maltose neopentyl glycol (LMNG; Anatrace). After centrifugation at 48,000*g* for 45 min, the supernatant was collected and subjected to affinity chromatography with Strep-Tactin resin (IBA). The resin was then rinsed with wash buffer (buffer W) containing 20 mM Hepes (pH 7.5), 150 mM NaCl, 10% glycerol, and 0.04% GDN. The protein was eluted with buffer W supplemented with 5 mM d-desthiobiotin. The protein was then concentrated and further purified by size exclusion chromatography using Superose 6 Increase 10/300 GL (GE Healthcare) preequilibrated in buffer containing 20 mM Hepes (pH 7.5), 150 mM NaCl, and 0.02% GDN. The peak fractions were pooled and concentrated to approximately 15 mg ml^−1^ for cryo-sample preparation.

Truncated p97 (p97^Δ^) with the deletion of amino acid residues 1 to 20 was cloned downstream of the p10 promoter, while truncated Derlin-1 (Der^Δ^) with the deletion of amino acid residues 215 to 239 was cloned downstream of the polyhedrin promoter in the same one pFastBac-Dual vector, in which Der^Δ^ contains a PreScission Protease cleavage site and a C-terminal twin strep tag. The Der^Δ^-p97^Δ^ coexpression construct was transformed into DH10Bac *Escherichia coli* cells for bacmid preparation, which was isolated from 5 ml of overnight cultures by the magnetic separation method. Baculoviruses were prepared according to the manufacturer’s manual (Invitrogen, Bac-to-Bac). Sf9 cells (Expression Systems, 94-001) were cultured in protein-free ESF 921 Insect Cell Culture Medium (Expression Systems, 96-001) at 27°C. Der^Δ^-p97^Δ^ was coexpressed in Sf9 cells for 72 hours using baculoviruses before harvest.

For the purification of the truncated Der^Δ^-p97^Δ^ complex, the baculovirus-infected Sf9 cells were collected and resuspended in lysis buffer containing 20 mM Hepes (pH 7.5), 150 mM NaCl, 10% glycerol, 10 mM MgCl_2_, DNase I (0.2 mg ml^−1^), and 1× protease inhibitor (MCE). The purification procedures of the apo truncated Der^Δ^-p97^Δ^ complex were the same as for the full-length Derlin-1–p97 complex. Moreover, different ATP analogs (ADP·BeF_x_ or ATP·BeF_x_) were added through the whole purification process to obtain the Der^Δ^-p97^Δ^ complex at different states, starting from resuspending cells in lysis buffer to the final purification by size exclusion chromatography.

### Cryo-EM sample preparation and data collection

Three-microliter aliquots of the samples were applied to glow-discharged holey carbon grids (Quantifoil R1.2/1.3 Au, 300 mesh), and after 10 s of incubation, the grids were blotted for 1 s and rapidly plunged into liquid ethane cooled by liquid nitrogen, using a Vitrobot Mark IV (FEI) operated at 8°C and 100% humidity. The grids were imaged using a Titan Krios transmission electron microscope (FEI) operated at 300 kV, with the specimen maintained at liquid nitrogen temperatures. Images were automatically collected with EPU software (FEI) on a K3 camera (Gatan) operated in super-resolution counting mode, placed at the end of a GIF Quantum energy filter (Gatan), functioning in zero-energy-loss mode with a slit width of 15 eV. Data were typically collected at a nominal magnification of 81,000 (corresponding to a physical pixel size of 1.1 Å), with a defocus range between −1.0 and −2.5 μm. The dose rate was set to ~17.2 electrons/Å^2^ per second, and the total exposure time was 2.9 s, resulting in a total dose of 50 electrons/Å^2^, fractionated into 32 frames.

### Cryo-EM data processing

For the full-length WT Derlin-1–p97 dataset, a total of 9796 cryo-EM images were collected, and motion correction was performed on the dose-fractioned image stacks using MotionCor2 with dose weighting (*42*, *43*). The contrast transfer function (CTF) parameters of each image were determined with Gctf (*44*), and automatic particle picking was carried out using Gautomatch-v0.56 (https://www2.mrc-lmb.cam.ac.uk/download/gctf_gautomatch_cu10-1-tar-gz/) on the basis of templates generated from a few hundred manually picked particles. Subsequent image processing steps were performed with RELION-3.1 (*45*) and cryoSPARC (*31*). An overview of the data processing procedure is shown in fig. S2A. The particles were first extracted with 4× binning (4.4 Å/pixel), and then junk particles were removed by two rounds of 2D classifications. The remaining particles were then extracted with 2× binning (2.2 Å/pixel) and subjected to 3D classification with an initial reference generated by cryoSPARC. A total of 994,364 particles corresponding to the best two classes were reextracted without binning (1.1 Å/pixel) and further processed with 3D autorefinement and solvent-masked postprocessing, which generated a cryo-EM density map with an overall resolution of 4.5 Å. Finally, additional one round of 3D classification was performed to analyze the flexibility of Derlin-1 part.

For the Der^Δ^-p97^Δ^ apo dataset, a total of 22,808 cryo-EM images were collected. After motion correction and CTF estimation in RELION, automatic particle picking with Gautomatch was performed. An overview of the data processing procedure is shown in fig. S4A. The particles were first extracted with 4× binning (4.4 Å/pixel), and then junk particles were removed by two rounds of 2D classifications. The remaining particles were subjected to heterogeneous refinement, using four initial references generated by cryoSPARC. The particles corresponding to the best class were reextracted without binning (1.1 Å/pixel) and further processed with 3D autorefinement. Two rounds of sequential skip alignment 3D classifications were performed with the first-round mask on Derlin-1 and the second-round mask on Derlin-1 and four p97 NTDs, yielding a total of 183,316 particles, which generated a reconstruction with an overall resolution of 3.67 Å after 3D autorefinement, CTF refinement, Bayesian polishing, and nonuniform refinement with C1 symmetry imposed. In addition, after the first-round skip alignment 3D classification, a total of 694,019 good particles were subjected to 3DVA in cryoSPARC (*36*). Meanwhile, the 694,019 good particles were also subjected to a skip alignment 3D classification with a mask on the whole p97 hexamer.

For the Der^Δ^-p97^Δ^/ADP·BeF_x_ dataset, a total of 7,052 cryo-EM images were collected. After motion correction and CTF estimation in RELION, automatic particle picking with Gautomatch was performed. An overview of the data processing corresponding to this dataset is shown in fig. S6A. Briefly, the particles were first extracted with 4× binning, and junk particles were removed by two rounds of 2D classifications. The remaining particles were then subjected to heterogeneous refinement in cryoSPARC. The particles corresponding to the best class were reextracted without binning and further processed with 3D autorefinement. Two rounds of skip alignment 3D classifications were performed with the mask on Derlin-1, yielding a total of 248,684 particles, which generated a map with an overall resolution of 3.61 Å after 3D autorefinement, CTF refinement, Bayesian polishing, and nonuniform refinement with C1 symmetry imposed.

For the Der^Δ^-p97^Δ^/ATP·BeF_x_ dataset, a total of 10,883 cryo-EM images were collected. After motion correction and CTF estimation in RELION, automatic particle picking with Gautomatch was performed. An overview of the data processing corresponding to this dataset is shown in fig. S8A. In short, the particles were first extracted with 4× binning, and junk particles were removed by two rounds of 2D classifications. The remaining particles were then subjected to heterogeneous refinement with six initial references generated by cryoSPARC. One class showed a similar shape to the Der^Δ^-p97^Δ^/ADP·BeF_x_ complex with a total of 974,202 particles. These particles were reextracted without binning and further processed with 3D autorefinement. The skip alignment 3D classification with the mask on Derlin-1 and four p97 NTDs were then performed, yielding a total of 307,745 particles, which generated a map with an overall resolution of 3.65 Å after 3D autorefinement, CTF refinement, Bayesian polishing, and nonuniform refinement with C1 symmetry imposed. Moreover, another class corresponding to the openDer^Δ^-p97^Δ^/ATP·BeF_x_ conformation was discovered after heterogeneous refinement, with a total of 959,419 particles. Then, these particles were reextracted without binning and further processed with 3D autorefinement. Two rounds of skip alignment 3D classifications with the mask on Derlin-1 were performed, yielding a total of 62,905 particles, which generated a map with an overall resolution of 4.51 Å after 3D autorefinement, CTF refinement, Bayesian polishing, and nonuniform refinement with C1 symmetry imposed.

Local resolution estimation was performed by cryoSPARC, and all the resolutions were estimated with the gold standard Fourier shell correlation 0.143 criterion with high-resolution noise substitution (*46*–*48*).

### Model building and refinement

The cryo-EM structure models of human Derlin-1 (PDB 7CZB) and human p97 (PDB 5FTK and 5FTN) and the crystallographic structure model of SHP box (PDB 5GLF) were used as references for initial model building of Der^Δ^-p97^Δ^ apo and Der^Δ^-p97^Δ^/ADP·BeF_x_ using Phenix (*49*). The initial models were then docked into the electron density map using Chimera (*50*), followed by iterative manual adjustment in COOT (*51*), and real-space refinement using Phenix. For openDer^Δ^-p97^Δ^/ATP·BeF_x_ data, the structure of Der^Δ^-p97^Δ^/ADP·BeF_x_ was used as initial model and the entire bodies of individual Derlin-1 protomers were used separately in building the structure model for the Derlin-1 part in open state.

### Lentivirus and infection

For lentiviral transduction, p97 short hairpin RNAs (shRNAs) were cloned into the pLKO.1 lentivector. The shRNA sequences are as follows: the oligonucleotide is 5′- CCGGGAATAGAGTTGTTCGGAATTTCTCGAGAAATTCCGAACAACTCTATTCTTTTTG-3′ (forward) and 5′- AATTCAAAAAGAATAGAGTTGTTCGGAATTTCTCGAGAAATTCCGAACAACTCTATTC-3′ (reverse). The shRNA plasmid was constructed according to the instructions for the pLKO.1 plasmid. The lentivirus package was also prepared according to the pLKO.1 standard protocol. Transduced human *Derlin-1* knockout (KO) human embryonic kidney (HEK) 293T cells with the lentivirus were selected using puromycin (3 μg/ml).

### Cell culture, transfections, affinity capture, and immunoblotting

HEK293T cells were cultured in Dulbecco’s modified Eagle’s medium (Sigma-Aldrich) supplemented with 10% fetal bovine serum (FBS) (Wisent Corporation, catalog no. 080-150) at 37°C with 5% CO_2_. Human *Derlin-1* KO HEK293T cell line was produced with CRISPR-Cas9 and confirmed with genomic DNA genotyping-PCR and Western blot.

Cells were cotransfected with 2.67 μg of pcDNA3.4-NHK with a C-terminal flag tag, 2.67 μg of no-tagged pcDNA3.4-p97 encoding WT or mutants or the empty vector, and 2.67 μg of modified pcDNA3.4-Derlin-1 with a C-terminal twin strep tag encoding WT or mutants or the empty vector per 6-cm dish using Lipofectamine 2000 (Thermo Fisher Scientific), according to the manufacturer’s instructions, and incubated for 6 hours at 37°C. Subsequently, transfection medium was replaced with fresh culture medium, and cells were incubated at 37°C for 48 hours before harvest. For p97 knockdown cells, *Derlin-1* KO cells infected with lentivirus for 2 days were cotransfected with plasmids and harvested after 48 hours. HEK293T cells were collected by centrifugation and washed with phosphate-buffered saline (PBS). The entire purification process was carried out at 4°C. Cells were resuspended in 1 ml of buffer containing 20 mM Hepes (pH 7.5), 150 mM NaCl, 10% glycerol, 10 mM MgCl_2_, DNase I (0.5 mg ml^−1^), and 1× protease inhibitor (MCE) and sonicated for 20 s on ice, and then the samples were solubilized at 4°C for 3 hours with 1.5% (w/v) n-Dodecyl-β-D-Maltoside (DDM) (Anatrace). After centrifugation at 16,900*g* for 20 min, the supernatant was collected and applied to Strep-Tactin XT superflow resin (IBA). The beads were rinsed with wash buffer containing 20 mM Hepes (pH 7.5), 150 mM NaCl, 10% glycerol, and 2 mM DDM. The beads with strep-tagged proteins were resuspended in loading buffer and then run on uniform SDS-PAGE gels before being transferred to polyvinylidene difluoride membranes for immunoblotting.

### RNA extraction and RT-qPCR assay

Total RNA extraction and cDNA synthesis were conducted using the FastPure Cell /Tissue Total RNA Isolation Kit V2 (Vazyme) and Hifair III 1st Strand cDNA Synthesis SuperMix for qPCR Kit (Vazyme) according to the manufacturer’s instructions. Reverse transcription qPCR (RT-qPCR) was performed with Hieff qPCR SYBR Green Master Mix (Yeasen) on the Roche LightCycler 480 Real-Time PCR System. Subsequently, the relative mRNA expression of NHK was calculated using the 2^−ΔΔCT^ method (*52*). The primer sequences were as follows: NHK, 5′-GATCAACGATTACGTGGAGAAGG-3′ (forward) and 5′-CCTAAACGCTTCATCATCATACGCA-3′ (reverse); actin, 5′- CACCATTGGCAATGAGCGGTTC-3′ (forward) and 5′-AGGTCTTTGCGGATGTCCACGT-3′ (reverse).

### Antibodies

The following antibodies were used: anti-NHK [SERPINA1 rabbit polyclonal antibody (pAb); ABclonal, A1015], anti–Derlin-1 (Derlin-1 rabbit pAb; Abcam, ab176732), anti–glyceraldehyde-3-phosphate dehydrogenase (GAPDH) (GAPDH mouse monoclonal antibody; ProteinTech, 60004-1-Ig), anti-p97 (VCP Rabbit pAb, ABclonal, A13368), anti-strep (Mouse anti Strep II-Tag mAb, ABclonal, AE066), anti-rabbit [ProteinFind Goat Anti-Rabbit IgG (H+L), HRP Conjugate, HS101-01], anti-mouse (Anti-mouse IgG, HRP-linked Antibody, Cell Signaling Technology, #7076).
