# Supplementary material for: The cryo-EM structure of the human ERAD retrotranslocation complex
Source: Sci Adv. 2023 Oct 13;9(41):eadi5656. doi: 10.1126/sciadv.adi5656 (PMC10575581; doi:10.1126/sciadv.adi5656)
Supplement: Supplementary file 1 — Figs. S1 to S12 Legend for movie S1 [file sciadv.adi5656_sm.pdf]

Science Advances
AAAS

Supplementary Materials for

The cryo-EM structure of the human ERAD retrotranslocation complex

Bing Rao *et al.*

Corresponding author: Yu Cao, yu.cao@shsmu.edu.cn

Sci. Adv. **9**, eadi5656 (2023)
DOI: 10.1126/sciadv.adi5656

The PDF file includes:

Figs. S1 to S12
Legend for Movie S1

Other Supplementary Material for this manuscript includes the following:

Movie S1

Supplementary Materials

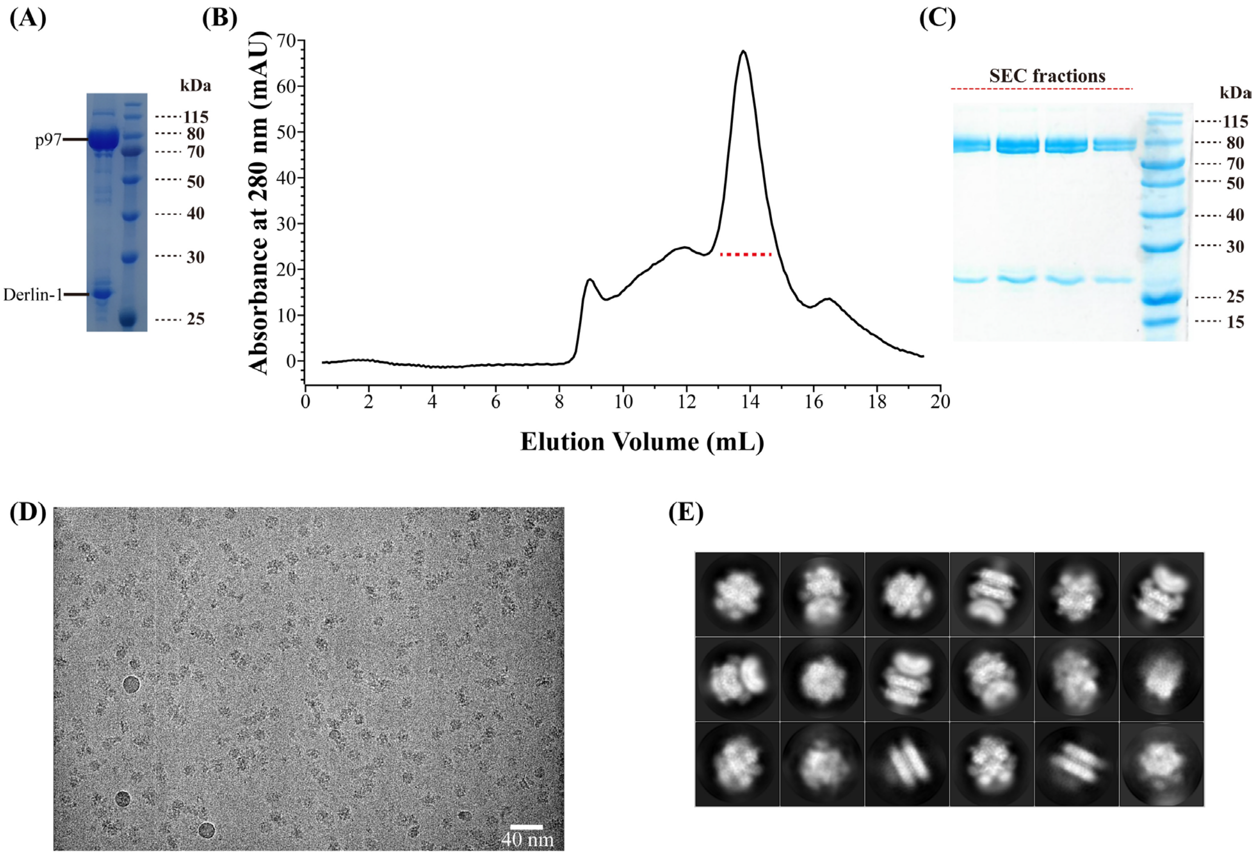

Fig. S1. Purification and characterization of the full-length human Derlin-1-p97 complex. (A): SDS-PAGE analysis of purified recombinant full-length human Derlin-1-p97 complex expressed in Expi293F cells. **(B):** Size-exclusion chromatography (SEC) profile of the full-length human Derlin-1-p97 complex. **(C):** SDS–PAGE analysis of further purified human Derlin-1-p97 complex corresponding to the peak fractions (indicated by red dashed lines) in b. **(D):** A representative cryo-EM micrograph image of the full-length human Derlin-1-p97 complex. **(E):** The representative 2D class averages of the full-length human Derlin-1-p97 complex.

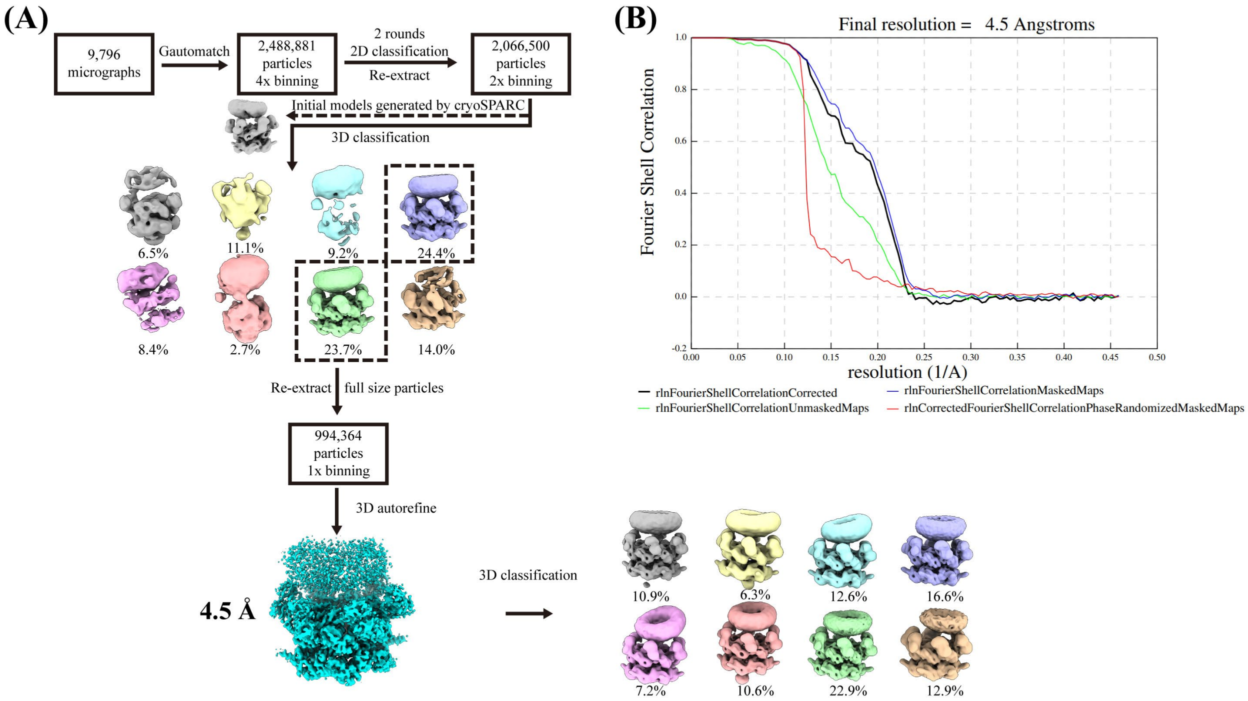

Fig. S2. Cryo-EM analysis of the full-length human Derlin-1-p97 complex. (A): The flow chart of cryo-EM data processing on full-length human Derlin-1-p97 complex. **(B):** The gold-standard Fourier shell correlation (FSC) curve of cryo-EM map of the full-length human Derlin-1-p97 complex.

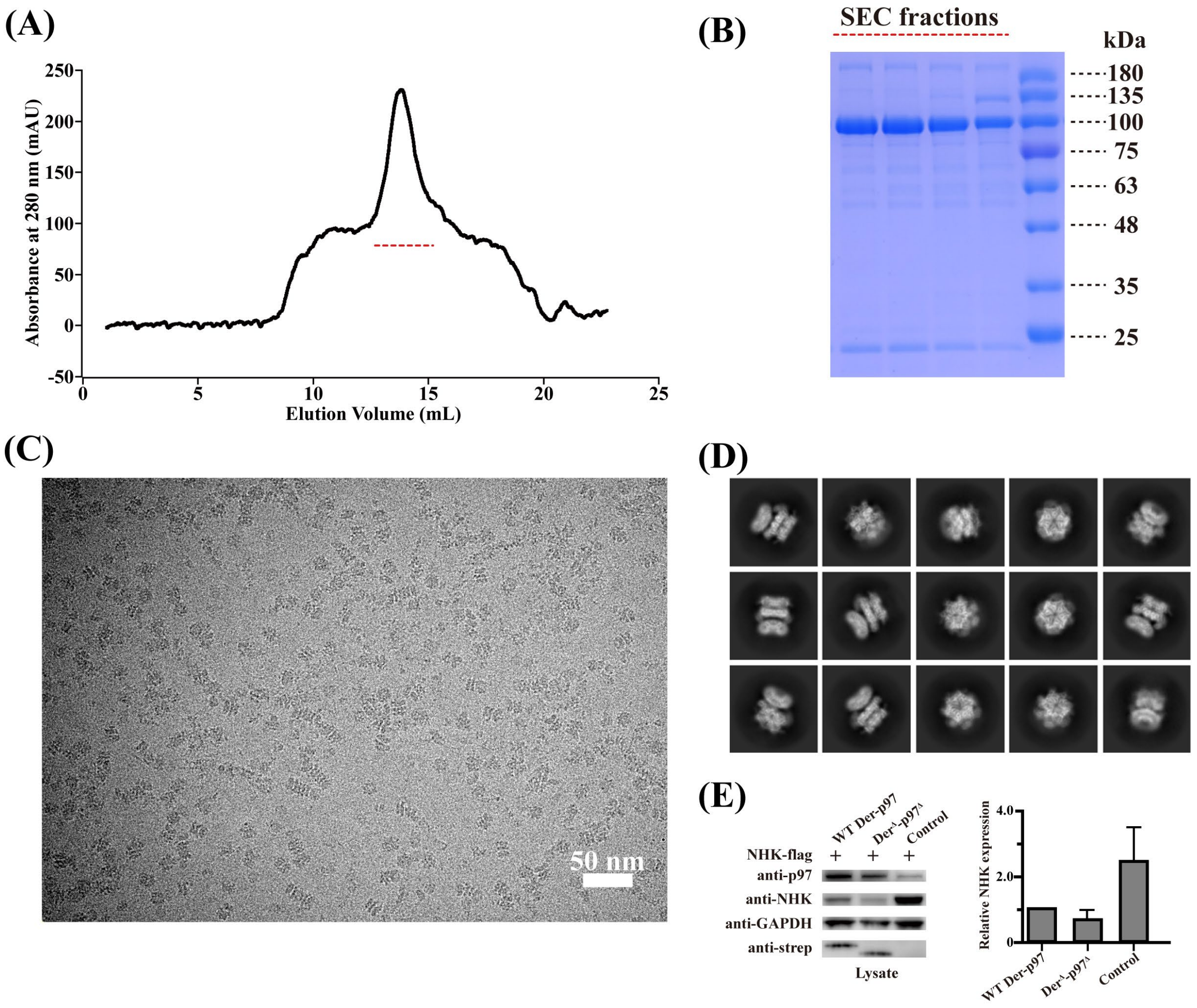

Fig. S3. Purification and characterization of the truncated human Derlin-1-p97 (Der$^{\Delta}$-p97$^{\Delta}$) apo complex. **(A):** SEC profile of the Der$^{\Delta}$-p97$^{\Delta}$ apo complex. **(B):** SDS–PAGE analysis of purified human Der$^{\Delta}$-p97$^{\Delta}$ apo complex corresponding to the peak fractions (indicated by red dashed lines) in a. **(C):** A representative cryo-EM micrograph image of the Der$^{\Delta}$-p97$^{\Delta}$ apo complex. **(D):** The representative 2D class averages of the Der$^{\Delta}$-p97$^{\Delta}$ apo complex. **(E):** Immunoblot of lysates of 293T Derlin-1 KO/p97 KD cells expressing NHK (control), co-expressing NHK, wild-type Derlin-1 with strep tag, and wild-type p97 (WT), and co-expressing NHK, Derlin-1$^{\Delta 215\text{-}239}$ with strep tag, and p97$^{\Delta 1\text{-}20}$ (Der$^{\Delta}$-p97$^{\Delta}$). **Left:** Representative Western blot results. **Right:** A semiquantitative analysis independently repeated three times. The WT band densities were respectively set as 1 and served as the normalization reference for the other bands in the same SDS-PAGE.

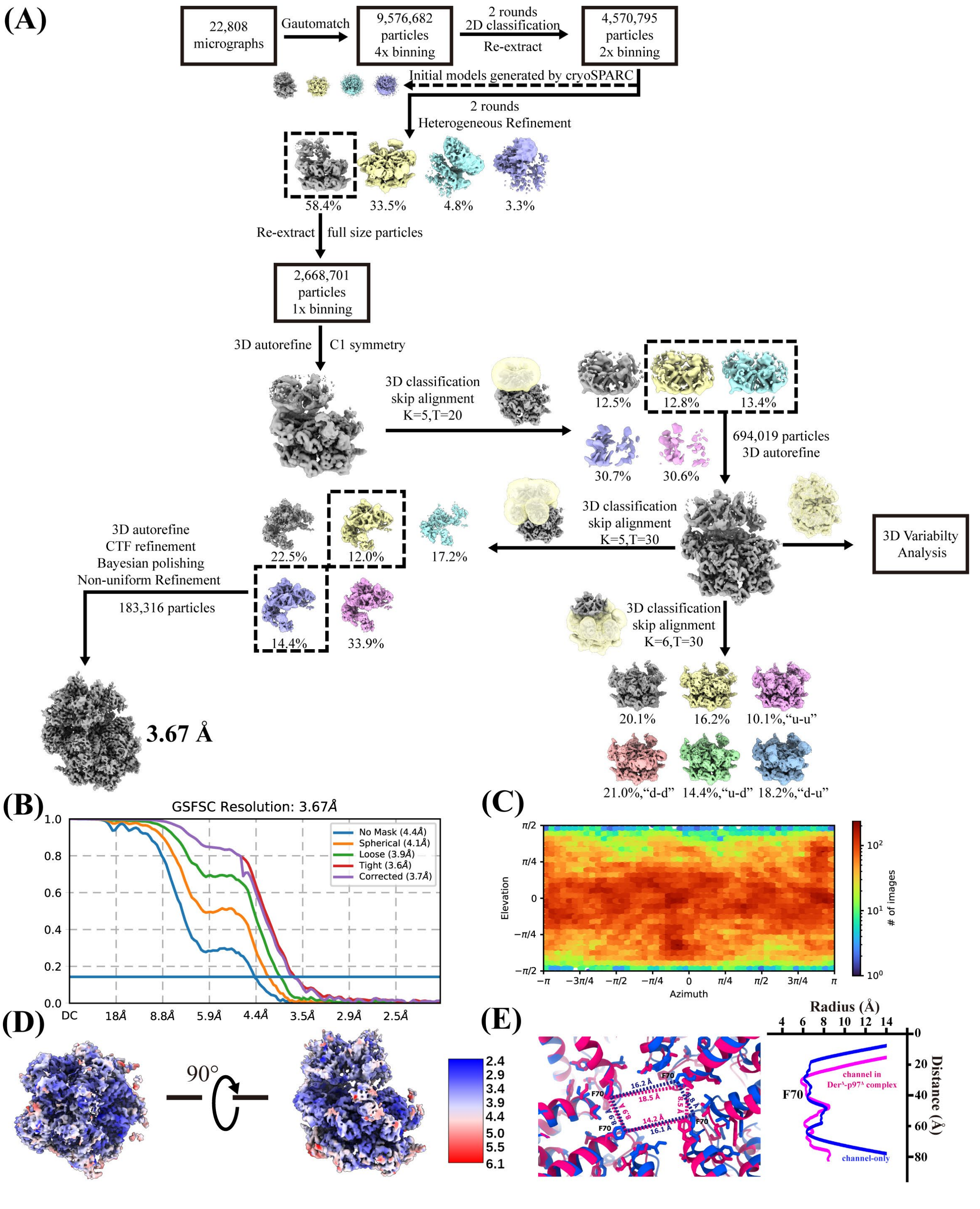

Fig. S4. Cryo-EM analysis of the Der$^{\Delta}$-p97$^{\Delta}$ apo complex. (A): The flow chart of cryo-EM data processing on the Der$^{\Delta}$-p97$^{\Delta}$ apo complex. **(B):** The gold-standard Fourier shell correlation (FSC) curve for the final cryo-EM map of the Der$^{\Delta}$-p97$^{\Delta}$ apo complex, generated by cryoSPARC with non-uniform refinement. **(C):** The distribution of orientations over azimuth and elevation angles for particles included in the calculation of the final map. **(D):** Local resolution maps were generated by cryoSPARC and shown as surface models in two orientations. **(E):** The structural superposition between the Derlin-1 central tunnel in Der$^{\Delta}$-p97$^{\Delta}$ apo complex (pink) and previously reported channel-only structure (blue, PDB ID 7CZB). The radius for the inner pathway calculated by HOLE program was shown in the right panel.

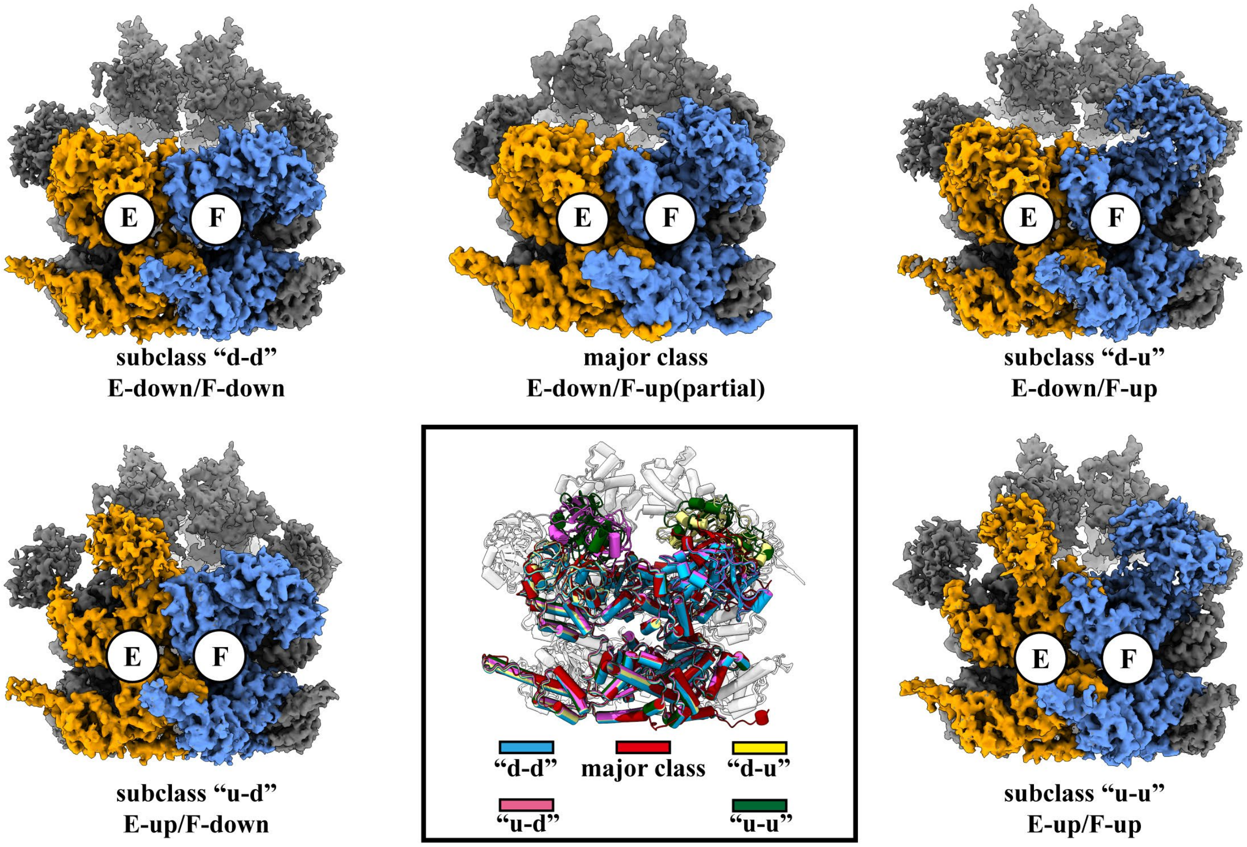

Fig. S5. The variability in the conformations of the unoccupied NTDs of the Der^{Δ}-$p97^{\Delta}$ apo complex. Five subclasses identified in the 3D classification on the particles of the Der^{Δ}-$p97^{\Delta}$ apo complex were shown as the surface models and designated as “d-d” (upper panel left), “d-u” (upper panel right), “u-d” (lower panel left), and “u-u” (lower panel right) based on the conformations of the NTDs of $p97^{E}$ and $p97^{F}$, as well as the major class (upper panel middle). The electron densities corresponding to the $p97^{E}$ and $p97^{F}$ were colored orange and blue, respectively, leaving the remaining density in gray. **In frame:** A structural superposition among the five subclasses of the Der^{Δ}-$p97^{\Delta}$ apo complex. The protomers E and F in each subclass were colored as the color scheme below, with all the other protomers in gray.

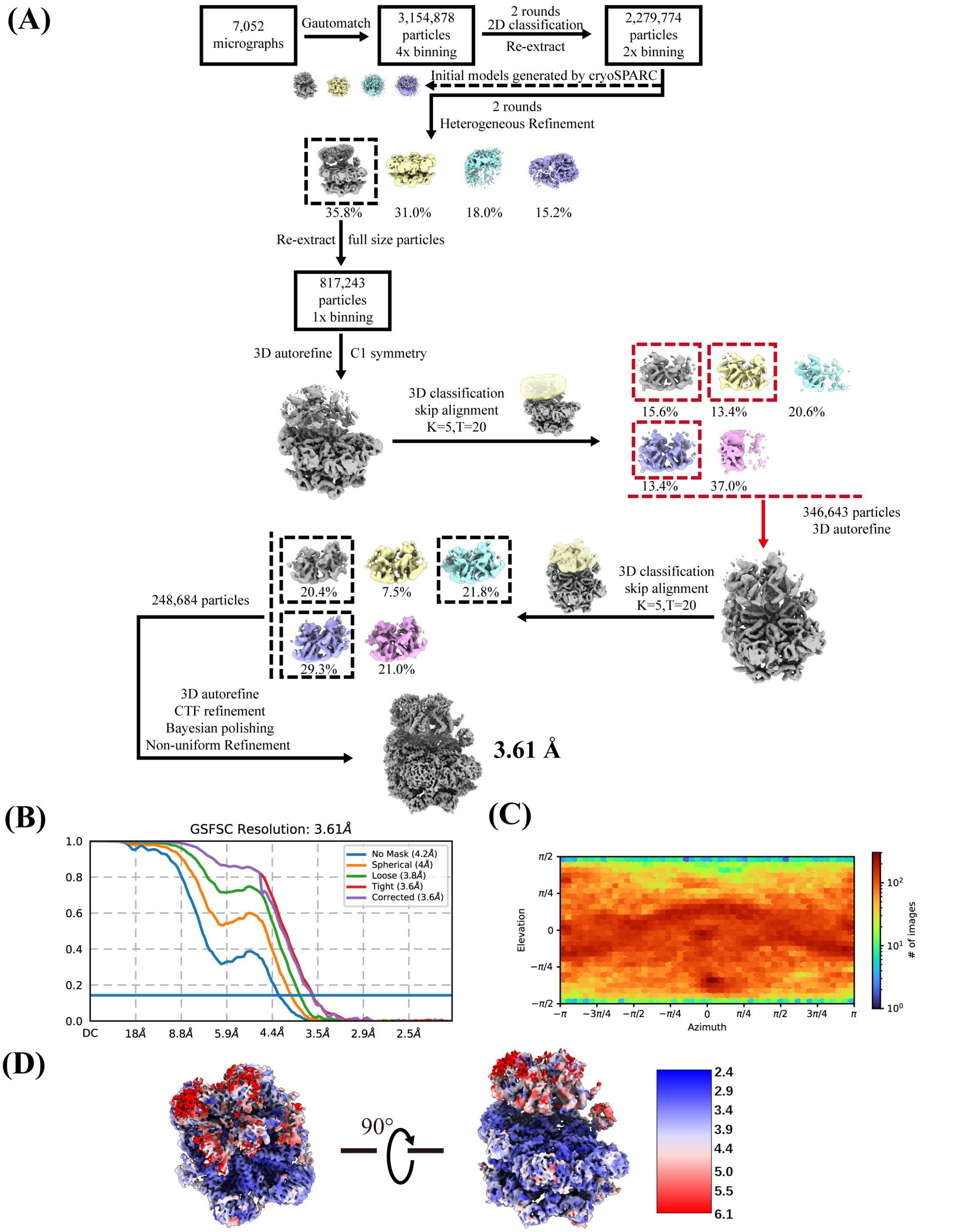

Fig. S6. Cryo-EM analysis of the Der$^{\Delta}$-p97$^{\Delta}$/ADP·BeF$_x$ complex. (A): The flow chart of cryo-EM data processing on the Der$^{\Delta}$-p97$^{\Delta}$/ADP·BeF$_x$ complex. **(B):** The gold-standard Fourier shell correlation (FSC) curve for the final cryo-EM map of the Der$^{\Delta}$-p97$^{\Delta}$/ADP·BeF$_x$ complex, generated by cryoSPARC with non-uniform refinement. **(C):** The distribution of orientations over azimuth and elevation angles for particles included in the calculation of the final map. **(D):** Local resolution maps were generated by cryoSPARC and shown as surface models in two orientations.

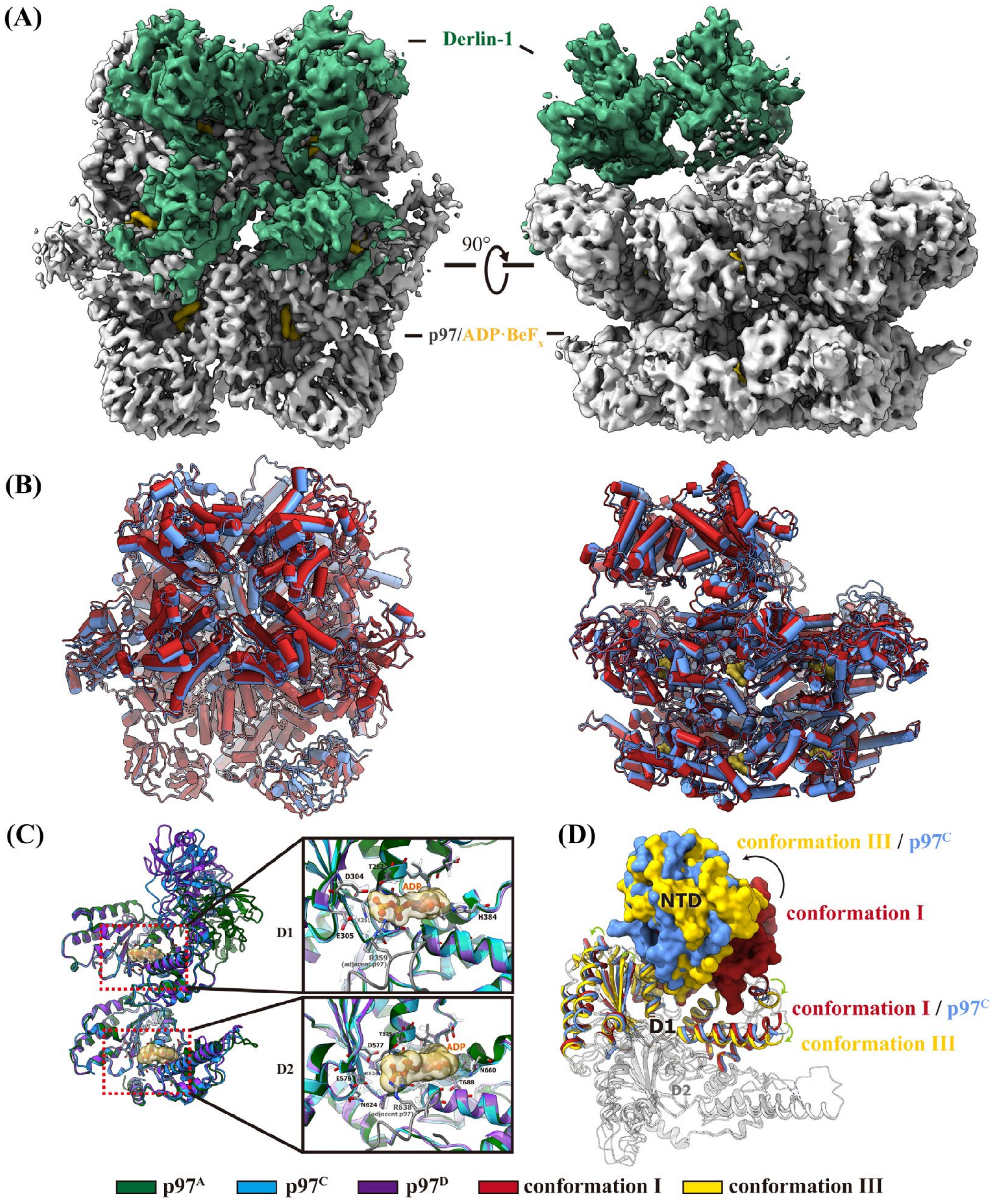

Fig. S7. The overall architecture of the decameric complex of the Der$^{\Delta}$-p97$^{\Delta}$/ADP·BeF_x complex. (A): The EM map of the Der$^{\Delta}$-p97$^{\Delta}$/ADP·BeF_x complex viewed from the lumenal side of the ER membrane (left) and parallel to the ER membrane (right). The cryo-EM map for the human Derlin-1 tetramer was colored green, the p97 hexamer was colored gray, and the ADP ligands were colored orange. **(B)**: The structural superposition between the Der$^{\Delta}$-p97$^{\Delta}$ apo and the Der$^{\Delta}$-p97$^{\Delta}$/ADP·BeF_x complexes. Both structures were shown as cartoon models and colored blue for the Der$^{\Delta}$-p97$^{\Delta}$ apo complex and red for the Der$^{\Delta}$-p97$^{\Delta}$/ADP·BeF_x complex, with the ligands shown as the orange surface models. **(C)**: The structural superposition among the p97 protomer A, C, and D. The D1 and D2 domains were zoomed in to show the details of ADP binding. Both the ADP and ADP-interacting residues in p97^{A} were shown as stick models and colored by elements, with the corresponding residues in p97$^{C/D}$ as transparent stick models. **(D)**: The structural superposition among the p97^{C} and p97 subunits in conformation I (PDB ID 5FTL) and III (PDB ID 5FTN), showing the conformation of p97^{C} was close to conformation I in its D1-D2 domain and close to conformation III in its NTD.

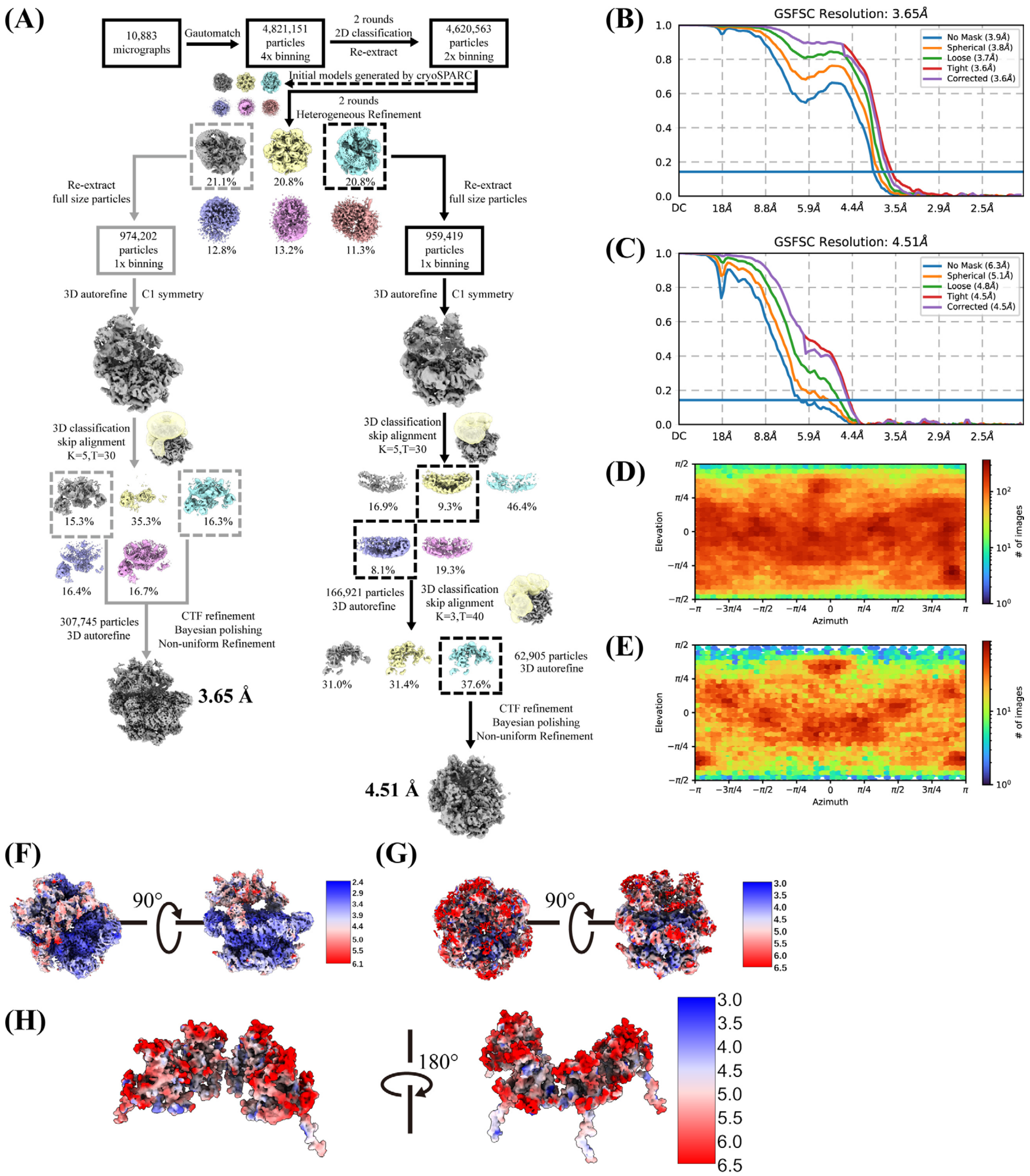

(A)
10,883 micrographs
Gautomatch
4,821,151 particles 4x binning
2 rounds 2D classification
Re-extract
4,620,563 particles 2x binning
Initial models generated by cryoSPARC
2 rounds Heterogeneous Refinement
21.1%
20.8%
20.8%
12.8%
13.2%
11.3%
Re-extract full size particles
Re-extract full size particles
974,202 particles 1x binning
959,419 particles 1x binning
3D autorefine
C1 symmetry
3D autorefine
C1 symmetry
3D classification skip alignment K=5,T=30
3D classification skip alignment K=5,T=30
15.3%
35.3%
16.3%
16.4%
16.7%
16.9%
9.3%
46.4%
8.1%
19.3%
307,745 particles 3D autorefine
CTF refinement Bayesian polishing Non-uniform Refinement
3.65 Å
166,921 particles 3D autorefine
3D classification skip alignment K=3,T=40
31.0%
31.4%
37.6%
62,905 particles 3D autorefine
CTF refinement Bayesian polishing Non-uniform Refinement
4.51 Å
(B)
GSFSC Resolution: 3.65Å
No Mask (3.9Å)
Spherical (3.8Å)
Loose (3.7Å)
Tight (3.6Å)
Corrected (3.6Å)
DC 18Å 8.8Å 5.9Å 4.4Å 3.5Å 2.9Å 2.5Å
(C)
GSFSC Resolution: 4.51Å
No Mask (6.3Å)
Spherical (5.1Å)
Loose (4.8Å)
Tight (4.5Å)
Corrected (4.5Å)
DC 18Å 8.8Å 5.9Å 4.4Å 3.5Å 2.9Å 2.5Å
(D)
Elevation
Azimuth
# of images
(E)
Elevation
Azimuth
# of images
(F)
90°
2.4 2.9 3.4 3.9 4.4 5.0 5.5 6.1
(G)
90°
3.0 3.5 4.0 4.5 5.0 5.5 6.0 6.5
(H)
180°
3.0 3.5 4.0 4.5 5.0 5.5 6.0 6.5

Fig. S8. Cryo-EM analysis of the Der$^{\Delta}$-p97$^{\Delta}$/ATP·BeF$_x$ complex. (A): The flow chart of cryo-EM data processing on the Der$^{\Delta}$-p97$^{\Delta}$/ATP·BeF$_x$ complex. **(B):** The gold-standard Fourier shell correlation (FSC) curve for the final cryo-EM map of the Der$^{\Delta}$-p97$^{\Delta}$/ATP·BeF$_x$ complex, which showed a similar conformation to the Der$^{\Delta}$-p97$^{\Delta}$/ADP·BeF$_x$ complex. **(C):** The gold-standard Fourier shell correlation (FSC) curve for the final cryo-EM map of the openDer$^{\Delta}$-p97$^{\Delta}$/ATP·BeF$_x$ complex. **(D):** The distribution of orientations over azimuth and elevation angles for particles corresponding to the map in (B). **(E):** The distribution of orientations over azimuth and elevation angles for particles corresponding to the openDer$^{\Delta}$-p97$^{\Delta}$/ATP·BeF$_x$ map in (C). **(F):** Local resolution maps corresponding to the map in (B) were generated by cryoSPARC and shown as surface models in two orientations. **(G):** Local resolution maps corresponding to the map in (C) were generated by cryoSPARC and shown as surface models in two orientations. **(H):** Local resolution maps of Derlin-1 in the openDer$^{\Delta}$-p97$^{\Delta}$/ATP·BeF$_x$ complex corresponding to the map in (C) were generated by cryoSPARC and shown as surface models in two orientations.

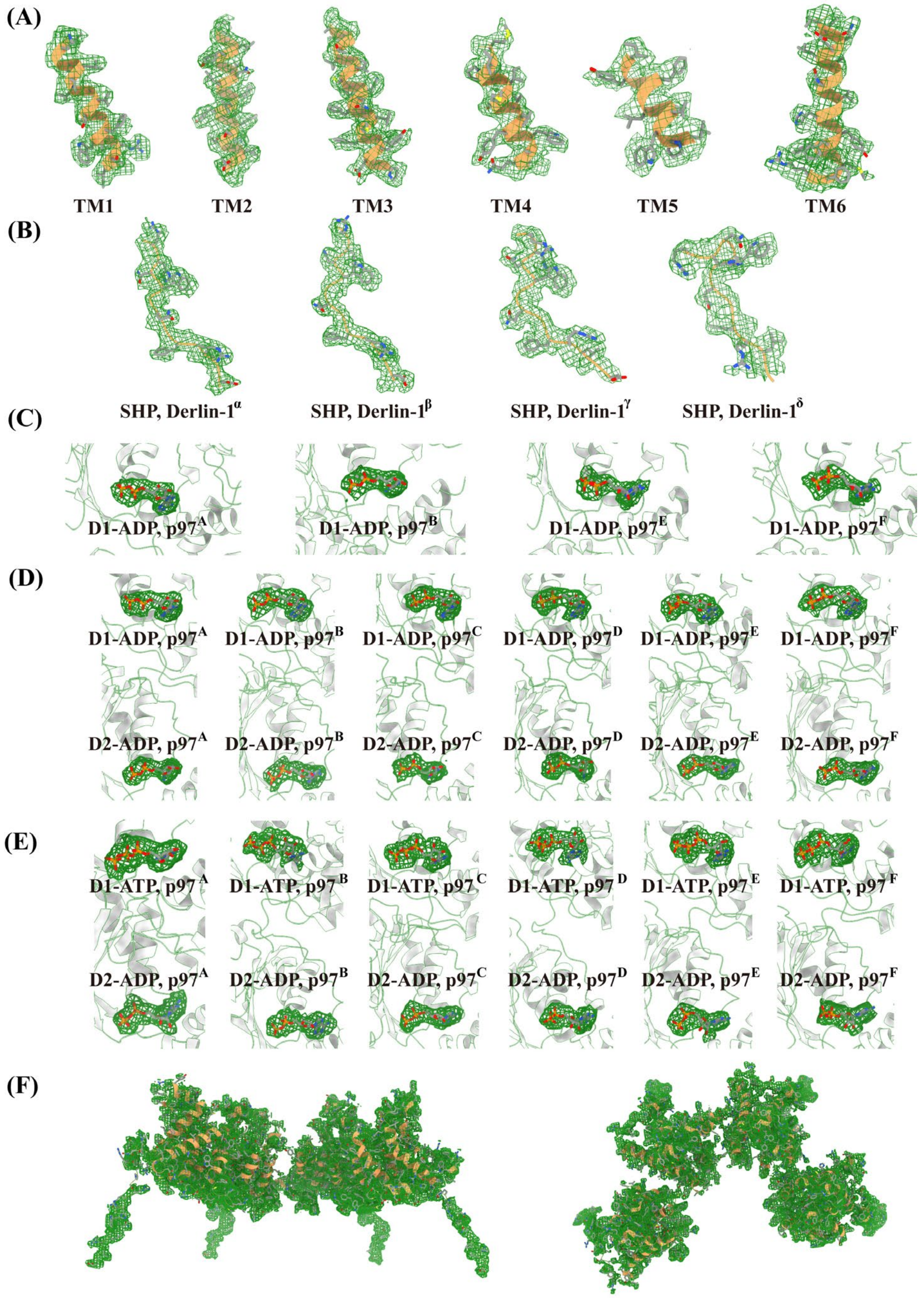

Fig. S9. The density and the model-fitting for the key secondary structures and ligands of the Der$^{\Delta}$-p97$^{\Delta}$ complex. (A): Cryo-EM maps and the models for transmembrane helices of Derlin-1$^{\beta}$ in the Der$^{\Delta}$-p97$^{\Delta}$ apo complex. **(B):** Cryo-EM maps and the models for the SHP boxes of Derlin-1$^{\alpha/\beta/\gamma/\delta}$ in the Der$^{\Delta}$-p97$^{\Delta}$ apo complex. **(C):** Cryo-EM maps for the ADP ligands bound in the D1 domain of the p97$^{A/B/E/F}$ in the Der$^{\Delta}$-p97$^{\Delta}$ apo complex. **(D):** Cryo-EM maps for the ADP ligands bound in the D1 and D2 domain of the p97 protomer A-F (from left to right) in the Der$^{\Delta}$-p97$^{\Delta}$/ADP·BeF$_x$ complex. **(E):** Cryo-EM maps for the ATP ligands bound in the D1 domain and the ADP ligands bound in the D2 domain of the p97 protomer A-F (from left to right) in the openDer$^{\Delta}$-p97$^{\Delta}$/ATP·BeF$_x$ complex. **(F):** Cryo-EM maps and the models for Derlin-1 in the openDer$^{\Delta}$-p97$^{\Delta}$/ATP·BeF$_x$ complex. All the maps were contoured to the same level.

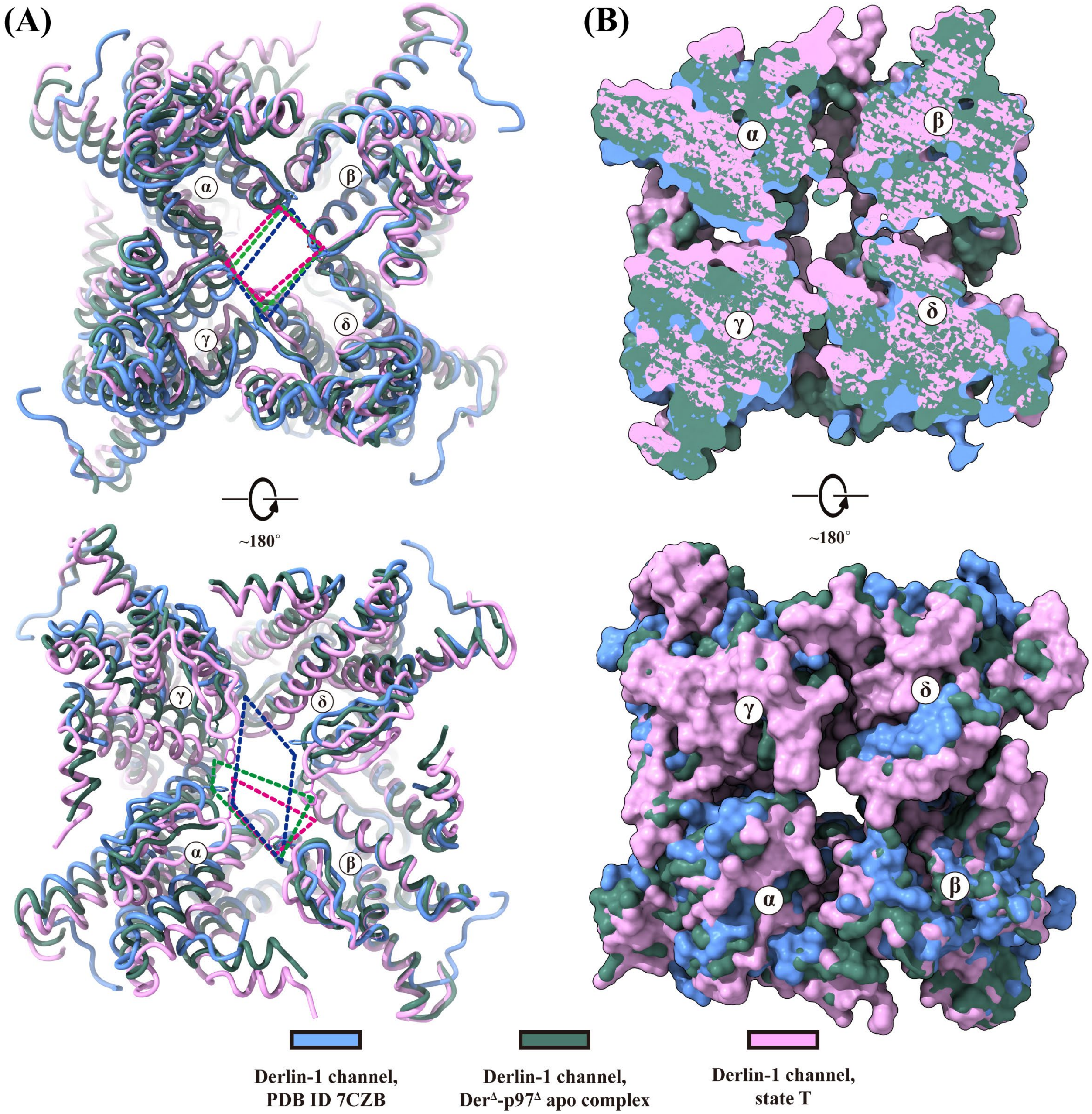

Fig. S10. The structural comparison among Derlin-1 structures in its channel-only state, Der$^{\Delta}$-p97$^{\Delta}$ apo complex, and state T. A structural superposition among the molecular models of the Derlin-1 tetramer from channel-only (blue), Der$^{\Delta}$-p97$^{\Delta}$ apo complex (green), and tightly closed state T (pink) in 3DVA was shown as cartoon models **(A)** and surface models **(B)**, with the protomer βs in three models aligned as the reference. The upper and lower panels show the superposition viewed from ER lumen and cytoplasmic side, with the key residues P66 (upper) and F146 (lower) shown as stick models and connected with dotted lines for a visual reference.

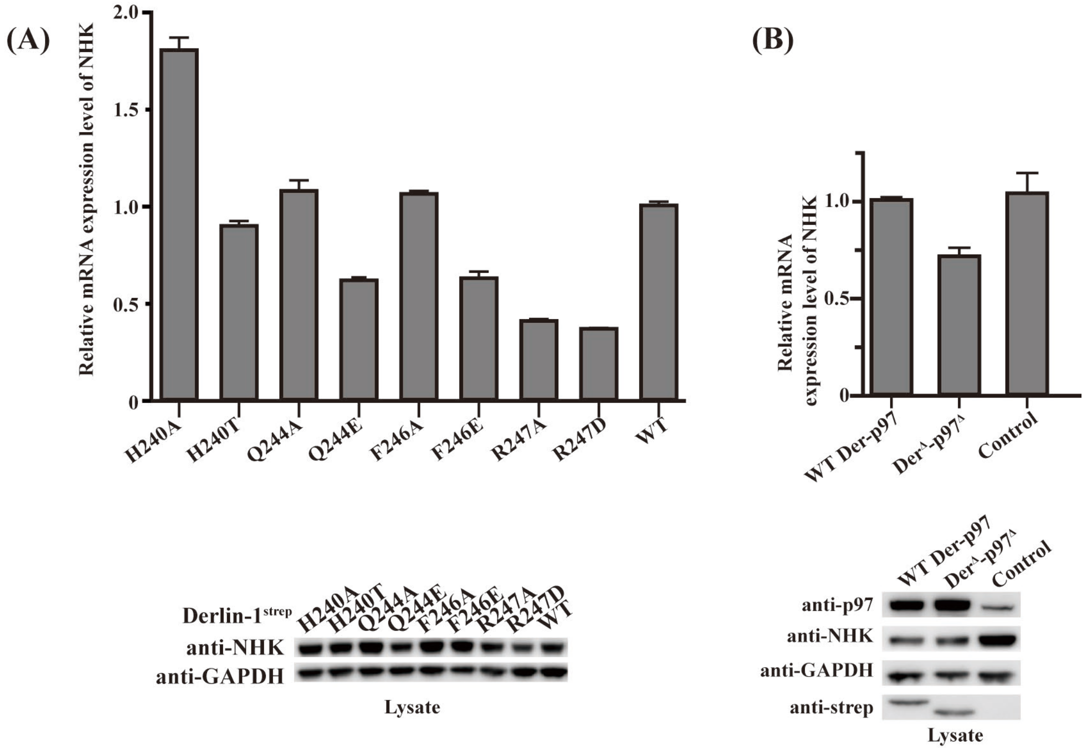

Fig. S11. qPCR analysis of the NHK levels. (A): Upper - the relative mRNA level of the NHK in 293T cells co-transfected with NHK, p97, and Derlin-1 carrying mutations as indicated below the data columns; lower - representative immunoblot results of lysates of 293T cells co-expressing NHK, strep-tagged Derlin-1, and p97 in qPCR analysis.

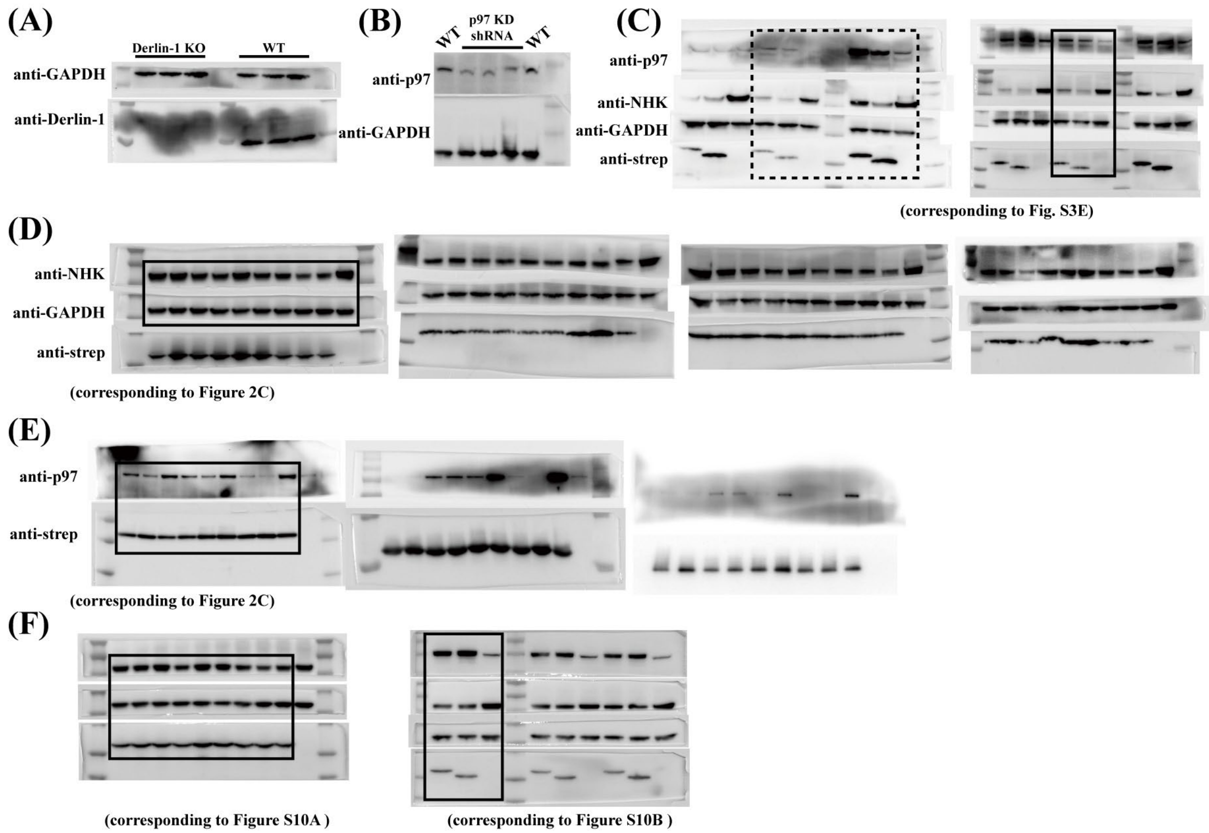

Fig. S12. Uncropped immunoblot images in this study. (A): Uncropped immunoblot images for the expression characterization of HEK293T Derlin-1 knockout cells. **(B):** Uncropped immunoblot images for the expression characterization of HEK293T p97 knockdown cells. **(C):** Uncropped immunoblot images corresponding to **Fig. S3E**. The black frame indicates the presented bands in the figures. The dotted frame indicates two sets of repeats used in statistics. **(D):** Uncropped immunoblot images corresponding to **Fig. 2C**. The black frame indicates the presented bands in the figures. **(E):** Uncropped immunoblot images corresponding to **Fig. 2C**. The black frame indicates the presented bands in the figures. **(F):** Uncropped immunoblot images corresponding to **Fig. S10**. The black frame indicates the presented bands in the figures.

Movie S1: The dynamic change of Der$^{\Delta}$-p97$^{\Delta}$ complex observed in 3DVA. The Der$^{\Delta}$-p97$^{\Delta}$ complex was shown as cartoon model and viewed parallel to the ER membrane (1st half of the video) and from the cytoplasmic side (2nd half of the video). The Derlin-1 channel was colored in blue but the N-terminal helix, loop L4, transmembrane helices TM1 and TM2, and the CTD of protomer γ were highlighted in pink, yellow, red, green, and orange, respectively. The p97 hexamer ring was colored in gray with the NTD in light cyan.
